# The oncofetal RNA-binding protein IGF2BP1 is a druggable, post-transcriptional super-enhancer of E2F-driven gene expression in cancer

**DOI:** 10.1093/nar/gkaa653

**Published:** 2020-08-06

**Authors:** Simon Müller, Nadine Bley, Bianca Busch, Markus Glaß, Marcell Lederer, Claudia Misiak, Tommy Fuchs, Alice Wedler, Jacob Haase, Jean Borges Bertoldo, Patrick Michl, Stefan Hüttelmaier

**Affiliations:** Institute of Molecular Medicine, Section for Molecular Cell Biology, Faculty of Medicine, Martin Luther University Halle-Wittenberg, 06120 Halle, Germany; Institute of Molecular Medicine, Section for Molecular Cell Biology, Faculty of Medicine, Martin Luther University Halle-Wittenberg, 06120 Halle, Germany; Institute of Molecular Medicine, Section for Molecular Cell Biology, Faculty of Medicine, Martin Luther University Halle-Wittenberg, 06120 Halle, Germany; Institute of Molecular Medicine, Section for Molecular Cell Biology, Faculty of Medicine, Martin Luther University Halle-Wittenberg, 06120 Halle, Germany; Institute of Molecular Medicine, Section for Molecular Cell Biology, Faculty of Medicine, Martin Luther University Halle-Wittenberg, 06120 Halle, Germany; Institute of Molecular Medicine, Section for Molecular Cell Biology, Faculty of Medicine, Martin Luther University Halle-Wittenberg, 06120 Halle, Germany; Institute of Molecular Medicine, Section for Molecular Cell Biology, Faculty of Medicine, Martin Luther University Halle-Wittenberg, 06120 Halle, Germany; Institute of Molecular Medicine, Section for Molecular Cell Biology, Faculty of Medicine, Martin Luther University Halle-Wittenberg, 06120 Halle, Germany; Institute of Molecular Medicine, Section for Molecular Cell Biology, Faculty of Medicine, Martin Luther University Halle-Wittenberg, 06120 Halle, Germany; Institute of Molecular Medicine, Section for Molecular Cell Biology, Faculty of Medicine, Martin Luther University Halle-Wittenberg, 06120 Halle, Germany; Department of Internal Medicine I, Faculty of Medicine, Martin Luther University Halle/Wittenberg, 06120 Halle, Germany; Institute of Molecular Medicine, Section for Molecular Cell Biology, Faculty of Medicine, Martin Luther University Halle-Wittenberg, 06120 Halle, Germany

## Abstract

The IGF2 mRNA-binding protein 1 (IGF2BP1) is a non-catalytic post-transcriptional enhancer of tumor growth upregulated and associated with adverse prognosis in solid cancers. However, conserved effector pathway(s) and the feasibility of targeting IGF2BP1 in cancer remained elusive. We reveal that IGF2BP1 is a post-transcriptional enhancer of the E2F-driven hallmark in solid cancers. IGF2BP1 promotes G1/S cell cycle transition by stabilizing mRNAs encoding positive regulators of this checkpoint like E2F1. This IGF2BP1-driven shortening of the G1 cell cycle phase relies on 3′UTR-, miRNA- and m^6^A-dependent regulation and suggests enhancement of cell cycle progression by m^6^A-modifications across cancers. In addition to E2F transcription factors, IGF2BP1 also stabilizes E2F-driven transcripts directly indicating post-transcriptional ‘super’-enhancer role of the protein in E2F-driven gene expression in cancer. The small molecule BTYNB disrupts this enhancer function by impairing IGF2BP1-RNA association. Consistently, BTYNB interferes with E2F-driven gene expression and tumor growth in experimental mouse tumor models.

## INTRODUCTION

RNA-binding proteins (RBPs), including the IGF2 mRNA-binding protein (IGF2BP) family are crucial regulators of tumor and stem cell fate (1–3). CLIP (cross-linking immunoprecipitation) studies suggest a plethora of mostly overlapping IGF2BP target mRNAs (4,5). Despite promiscuous RNA-binding properties and distinct, partially oncofetal expression patterns, all IGF2BP paralogues show an ‘oncogenic’ potential in cancer (6,7). However, among IGF2BPs, only IGF2BP1 shows strong conservation of oncogenic potential in cancer-derived cell lines (8,9). This was largely attributed to the inhibition of MYC mRNA decay by IGF2BP1 (10). This regulation, however, is an exception, since all IGF2BPs impair MYC mRNA turnover due to hindering cleavage by endonucleases in the coding region of MYC (11,12). The main role of IGF2BP1 in cancer cells is the impairment of miRNA/RISC-directed mRNA decay by safe-guarding target mRNAs in cytoplasmic mRNPs (8,13–15). Recently, IGF2BPs were identified as m^6^A-readers, associating preferentially with N^6^-methyladenosine modified target mRNAs (12). Validated for two mRNAs, MYC and SRF, m^6^A-enhanced mRNA association of IGF2BPs results in elevated mRNA stabilization and enforced expression of MYC and SRF, respectively (12,16). Despite consistent stimulation of tumor cell proliferation and tumor growth by IGF2BP1, conserved effector pathways remained unknown. Here, we reveal that IGF2BP1 stabilizes E2F1–3 mRNAs leading to enhanced E2F-driven gene expression and cell cycle progression in cancer cells. E2F-dependent regulation is frequently deregulated in cancer and tightly linked to the control of self-renewal versus differentiation potential of pluripotent stem cells (17,18). In cancer as well as progenitor cells, E2F expression is subjected to largely conserved regulation by various microRNAs (17,19). Surprisingly, regulation of E2F expression by RBPs was only reported for pumilio proteins (20). PUM1 and 2 were shown to impair E2F3 mRNA translation and promote miRNA-directed silencing of E2F3 expression in cancer cells, suggesting a rather tumor-suppressive role of both RBPs. In contrast, IGF2BP1 is considered to act in an oncogenic manner. Accordingly, a small molecule inhibitor of the protein, termed BTYNB (21), was recently reported. BTYNB was shown to impair the association of IGF2BP1 with the MYC RNA *in vitro* and 2D proliferation of various tumor cells. However, if BTYNB also interferes with other, conserved effector pathways of IGF2BP1 in cancer cells and impacts tumor growth remained largely elusive.

## MATERIALS AND METHODS

### Animal handling and ethics approvals

Immunodeficient athymic nude mice (FOXN1^nu/nu^) were obtained from Charles River. Animals were handled according to the guidelines of the Martin Luther University. Permission was granted by a local ethical review committee. For subcutaneous xenograft assays 1 × 10^5^ iRFP-labeled ES-2 cells or 2.5 × 10^5^ iRFP-labeled A549 cells (stably transduced using iRFP encoding lentiviruses) were harvested in media supplemented with 50% (v/v) matrigel (Sigma) and injected into the left flank of six-week old female immunodeficient athymic nude mice. For intraperitoneal assays 1 × 10^5^ iRFP-labeled ES-2 cells were harvested in PBS and injected into six-week old female immunodeficient athymic nude mice. Mice were held with access to chlorophyll-free food to avoid background noise in iRFP image acquisition. Subcutaneous tumor growth and volume were measured and monitored by non-invasive near-infrared imaging using a Pearl Trilogy Imaging System (LI-COR). Tumor volume was calculated using the formula 0.52 × *L*_1_ × *L*_2_ × *L*_3_. The mice were sacrificed, once the first tumor reached a diameter of 1.5 cm. For monitoring intraperitoneal tumor growth, isofluran-anaesthetized mice were weekly monitored by near-infrared imaging. Intraperitoneal fluorescence intensity of iRFP-labeled cells was quantified using the Image Studio software (LI-COR). Where indicated, ES-2 cells were pre-incubated with DMSO or 5 μM BTYNB for 24 h prior to injection, in suspension containing DMSO or BTYNB, into athymic nude mice. Prior injection, viable cells were counted using trypan blue and a TC20 Cell Counter (Bio-Rad).

### Cell cycle analyses

For cell cycle analyses, cells were harvested with trypsin (72 h post-transfection or otherwise indicated), fixed overnight in 70% ethanol at −20°C. DNA was stained with propidium iodide (Miltenyi Biotec; diluted 1:1000) at 37°C for 30 min in PBS supplemented with RNAse A (2 μg/ml; Sigma Aldrich) to deplete RNA. The DNA content was measured by flow cytometry using a MACS Quant Analyzer (Miltenyi Biotec) and analyzed using FlowJo. The FUCCI system was used to analyze the length of cell cycle phases. ES-2 cells, stably transduced with IncuCyte^®^ Cell Cycle Red/Green Lentivirus Reagent (Sartorius), were transfected with indicated siRNAs. Cells in the G2/M phase were enriched by FACS based on their green fluorescence using a FACS Melody sorter (BD Bioscience) 24 h post-transfection. Cell cycles phases were monitored based on their fluorescence using an IncuCyte S3 (Sartorius) starting immediately after sorting. Cell segmentation and quantification was performed using the Cell-By-Cell module (IncuCyte S3; Sartorius). Single cell tracking was subsequently processed using ImageJ.

### Cell culture and transfections

HepG2 (ATCC, RRID: CVCL_0027), A549 (ATCC, RRID: CVCL_0023), ES-2 (ATCC, RRID: CVCL_3509), MV3 (RRID: CVCL_W280) and PANC-1 (ATCC, RRID: CVCL_0480) and HEK293T/17 (ATCC, RRID:CVCL_1926) were cultured in Dulbecco's modified Eagle's medium (DMEM) supplemented with 10% fetal bovine serum (FBS) at 37°C and 5% CO_2_.

Transfection of cells with DNA or siRNAs was performed using Lipofectamine 3000 or Lipofectamine RNAiMAX (Thermo Fisher Scientific) according to the manufacturer's instructions. For the production of lentiviral particles 2.8 × 10^6^ HEK293T/17 cells were transfected using Lipofectamine 3000, the packaging plasmids psPax2 (Addgene: Plasmid #12260) and pMD2.G (Addgene: Plasmid #12259) and the lentiviral expression pLVX vector encoding iRFP, GFP, GFP-IGF2BP1 or GFP-IGF2BP1 KHmutant. For luciferase reporter studies 1 × 10^5^ cells were transfected using Lipofectamine 3000 and pmirGLO or NanoLuc plasmids. For genomic deletions via CRISPR/Cas9 5 × 10^5^ cells were transfected using Lipofectamine 3000, Cas9- and sgRNA-encoding plasmids (see CRISPR/Cas9 section). For the gene-specific depletion with siRNAs 5 × 10^5^ cells were transfected using 9 μl Lipofectamine RNAiMAX and 15nM siRNAs. Plasmids and siRNAs used are summarized in Supplementary Table S10.

The inhibitors BTYNB (Cayman Chemical) or Palbociclib (Selleckchem) were used at indicated concentrations. For RNA decay analyses, cells were treated with actinomycin D (5 μM, Sigma Aldrich) for indicated time points 72 h upon transfection.

### Lentiviral transduction

Lentiviral particle-containing supernatants were collected 24 and 48 h upon transfection of HEK293T/17 cells. Titers were analyzed 48 h post-infection of HEK293T/17 cells and determined by flow cytometry (GFP or iRFP) using a MACS Quant Analyzer (Miltenyi BioTec). Lentiviral transduction for downstream experiments was accomplished at 10 MOI (multiplicity of infection).

### CRISPR/Cas9-mediated genomic deletions

For the CRISPR/Cas9-mediated genomic deletions in the *IGF2BP1* and *METTL3 loci*, A549 cells were transfected with two CRISPR sgRNA-encoding plasmids (IGF2BP1: psg_RFP_IGF2BP1_Ex6, psg_RFP_IGF2BP1_Ex7; METTL3: psg_RFP_METTL3_Ex3–1, psg_RFP_METTL3_Ex3–2) and a Cas9 nuclease-encoding plasmids (pcDNA_Cas9_T2A_GFP). For the genomic deletion of the E2F1 3′UTR locus, PANC-1 cells were transfected with two CRISPR sgRNA-encoding (psg_RFP_E2F1_3p1, psg_RFP_E2F1_3p2, encoding sgRNAs targeting the last exon of E2F1 downstream of the stop-codon and upstream of the polyA-signal) and a Cas9 nuclease-encoding plasmids (pcDNA_Cas9_T2A_GFP). Single cell clones were generated by seeding one RFP- and GFP-positive cell per well using a FACS Melody sorter (BD Bioscience) 48 h post-transfection. The deletion of IGF2BP1 and METTL3 was validated by western blotting. The bi-allelic deletion of the E2F1 3′UTR in the *E2F1* gene locus was validated by PCR on isolated genomic DNA of single cell clones. CRISPR sgRNAs, plasmids and PCR primer are summarized in Supplementary Table S10.

### Luciferase assays

The E2F1–3′UTR (NM_005225.3) was amplified on genomic DNA and cloned in the pmirGLO plasmid (Promega, pmirGLO_E2F1_3p). Dual-GLO Luciferase reporter analyses were performed according to manufacturer's protocols. Luciferase activities (Firefly and Renilla) were determined 48 h post-transfection of reporters. Reporters containing a minimal vector-encoded 3′UTR (MCS) served as normalization controls. For luciferase reporter studies on the E2F-transcriptional activity, four E2F binding elements were cloned upstream of a minimal, NanoLuc-driving promoter (Promega, pNL3.1_4xE2F). NanoLuc reporter analyses were performed according to manufacturer's protocols. Luciferase activities were determined 48 h post-transfection of reporters. Reporters containing a minimal promoter served as normalization controls.

### Plasmids and cloning

Cloning strategies including plasmids, oligonucleotides used for PCR and restriction sites are summarized in Supplementary Table S10. All constructs were validated by sequencing.

### RNA sequencing and differential gene expression

Libraries for RNA-sequencing (RNA-seq) were generated according to the manufacturer's instructions. For total RNA-seq, 1 μg of total RNA served as input for rRNA depletion using RiboCop v1.2 (Lexogen). The Ultra Directional RNA Library kit (NEB) was used for strand-specific library generation. Library preparation and sequencing was performed on an Illumina NextSeq 500 platform at the Deep Sequencing Group (TU Dreseden). For the preparation of small RNA-seq libraries, 50 ng of total RNA served as input using the NEXTflex Small RNA Library Prep Kit v3 (Bio Scientific). Sequencing was performed on the Illumina HighSeq 2000 platform at the Deep Sequencing Group (TU Dreseden). For mRNA-seq libraries, total RNA served as input for a polyA-enrichment using oligo dT beads. Library preparation and sequencing was performed by Novogene (Hong Kong) on an Illumina HiSeq platform. First, Low quality read ends as well as remaining parts of sequencing adapters were clipped off using Cutadapt (V 1.6). For total and small RNA-seq analyses reads were aligned to the human genome (UCSC GRCh37/hg19) using TopHat2 (V 2.0.13) or Bowtie2 (V 2.2.4), respectively. FeatureCounts (V 1.4.6) was used for summarizing gene-mapped reads. Ensembl (GRCh37.75) or miRBase (V 20) were used for annotations (see supplementary table T1A). Differentially gene expression (DE) was determined by edgeR (V 3.12) using TMM normalization, as described previously (8).

### Kaplan–Meier analyses

For survival analyses, Kaplan–Meier plots and Hazard ratios (HR) were determined using GEPIA 2 (http://gepia2.cancer-pku.cn/#survival) based on the expression status of indicated genes in TCGA data sets with median group cutoff.

### MicroRNA–Target predictions

miRWalk 2.0 (http://zmf.umm.uni-heidelberg.de/apps/zmf/mirwalk2/), (22) was used for the analysis of miRNA-targeting in the 3′UTR of the E2F1 transcript (NM: 0055225.3). The following databases were considered: miRWalk, miRDB, PITA, MicroT4, miRMap, RNA22, miRanda, miRNAMap, RNAhybrid, miRBridge, PICTAR2 and Targetscan (Supplementary Table S6).

### IGF2BP1-CLIP and m^6^A-RIP- data analysis

IGF2BP1 CLIP data were analyzed as previously described (8). In brief, peak genomic coordinates from publicly available IGF2BP1-CLIP data (4,5,23) were obtained from ENCODE (24), NCBI GEO (4) and CLIPdb (25), were mapped to all annotated genes (RefSeq hg19) using bedtools (26). For IGF2BP1-binding, the following number of datasets was considered: two PAR-CLIP (HEK293), two eCLIP (hESCs), two eCLIP (HepG2) and two eCLIP (K562). For the analysis of transcript-specific m^6^A-modification, m^6^A-RIP-seq data, performed in A549 cells, were considered and obtained from MeT-DB (V2.0, (27)).

### Gene expression and correlation analysis

We obtained gene-level RNA-seq read counts of TCGA primary tumor samples and GTEx V7 normal tissue via the GDC data portal (portal.gdc.cancer.gov) and the GTEx portal (gtexportal.org), respectively, for the indicated tumor cohorts. Differential gene expression was assessed using R/edgeR (pmid: 19910308) by applying TMM normalization. Respective tumor and normal tissue sample data were normalized together to avoid composition bias. CPM transformation was utilized to obtain normalized expression values. For correlation analyses, RNA-seq data sets for protein-coding genes were log_2_-(FPKM+1)-transformed and the Pearson correlation coefficient with IGF2BP1 was determined.

### Gene set enrichment analysis (GSEA)

Gene set enrichment analyses (GSEA) were performed on pre-ranked lists using the GSEA-software (V3.0, (28)) with MSigDB (V7.0, (29)) gene sets for Hallmarks and KEGG pathways. All protein-coding genes were ranked according to the correlation coefficient with IGF2BP1 in TCGA RNA-seq data or the fold change determined upon IGF2BP1 knockdown or knockout by RNA-seq.

### Cell proliferation, spheroid, self-renewal and clonogenic assays

For the assessment of cell proliferation in 2D culture systems, 2.5 × 10^4^ cells were plated 24 h upon transfection and the amount of cells as well as propidium iodide-negative/-positive cells were determined by flow cytometry at indicated time points using a MACS Quant Analyzer (Miltenyi Biotec). In addition, cell confluency and vitality were determined by using an IncuCyte S3 system (Sartorius) with 10× magnification and CellTiter Glo (Promega) according to manufacturer's protocols. For spheroid growth in 3D culture systems, 1 × 10^3^ cells were seeded in 96-well round-bottom ultra-low attachment plates (Corning) 24 h post-transfection. Spheroid formation was induced by centrifugation for 3 min at 300 g. Spheroid growth was monitored for five additional days by bright-field microscopy using an IncuCyte S3 system (Sartorius) with 10× magnification. Additionally, cell viability was determined by using CellTiter Glo (Promega). For anchorage-independent growth and self-renewal, 1 × 10^3^ cells were seeded 24 h post-transfection in a layer of soft agar mixed with cell culture medium (0.35% agar) on another layer of soft agar containing a higher concentration (0.5% agar). Growth and colony formation were monitored for 14 days with medium exchange every 3 days, as described previously (30). Colonies were stained using MTT (Sigma-Aldrich). The number of colonies was determined by using the 2D Colony Analyzer tool of the MiToBo package for the Fiji software (http://fiji.sc). For clonogenic assays, 200 cells were seeded in six-well plates 24 h post-transfection. Colony formation was analyzed 14 days upon seeding. Colonies were stained by using 0.01% crystal violet for 60 min. Number of colonies were determined by using the 2D Colony Analyzer tool of the MiToBo package for the Fiji software.

### Drug synergy matrix screen

For the analysis of synergy between BTYNB and Palbociclib, the viability of ES-2 cells was determined 72 h upon drug exposure using CellTiter GLO in a drug matrix screen at indicated concentrations. Synergy relief maps were generated using the SynergyFinder web application (https://synergyfinder.fimm.fi, (31)) and the ZIP (Zero interaction potency) method.

### RNA isolation and RT-q-PCR

Total RNA from cell line experiments was isolated by using TRIzol. RNA integrity was determined on a Bioanalyzer 2100 (Agilent). For cDNA synthesis, two μg total RNA served as a template using M-MLV Reverse Transcriptase (Promega) and random hexamer primers following manufacturer's protocols. qPCR analysis was performed using a LightCycler 480 II (Roche) with the ORA™ qPCR Green ROX L Mix (highQu) using following PCR reactions: 5 min / 95°C, 45 cycles of 10 s/95°C, 10 s/60°C and 20 s/72°C. Primer pairs spanning an exon/exon borders were selected using Primer Blast (https://www.ncbi.nlm.nih.gov/tools/primer-blast/). Sequences are summarized in Supplementary Table S10. Relative RNA abundance was determined by the ΔΔC_t_ method, as previously described (8).

### Nascent RNA capture

The Click-iT Nascent RNA Capture Kit (Thermo Fisher) was used for the purification of newly synthesized RNAs according to manufacturer's protocol. In brief, PANC-1 cells were transfected with control or IGF2BP1-directed siRNAs for 72 h. Cells were further incubated with 0.2 mM 5-ethynyl uridine (EU) for 4 h. Total RNA was prepared using TRIzol. 10 μg of total RNA served as input for the biotinylation of the EU-labeled RNA by click reaction using 1 mM biotin azide. 1 μg of biotinylated RNA served as input for the purification of nascent RNAs using Streptavidin T1 magnetic beads. Total RNA and purified nascent RNA served as templates for cDNA-synthesis and qPCR analysis.

### RNA co-Immunoprecipitation (RIP)

For RNA co-immunoprecipitations (RIP) cell extracts (1 × 10^7^ per condition) were prepared on ice using RIP buffer (10 mM HEPES, 150 mM KCl, 5 mM MgCl_2_, 0.5% NP40, pH 7.0). Cleared lysates were incubated with indicated antibodies (anti-IGF2BP1- or anti-AGO2-antibodies) and Protein G Dynabeads (Life Technologies) for 60 min at room temperature (RT). After three washing steps with RIP buffer, protein–RNA complexes were eluted by addition of 1%SDS and 65°C/10 min. Protein enrichment was analyzed by western blotting. Co-purified RNAs were extracted using TRIZOL and analyzed by RT-q-PCR.

### Western blotting

Infrared western blotting analyses were performed as previously described (8). In brief, total protein of harvested cells was extracted in lysis-buffer (50 mM Tris–HCl (pH 7.4), 50 mM NaCl, 2 mM MgCl_2_, 1% SDS) supplemented with protease and phosphatase inhibitor cocktails (Sigma-Aldrich). Protein expression was analyzed by Western blotting with indicated primary antibodies by using fluorescence-coupled secondary antibodies and an infra-red scanner (LICOR). Antibodies used are indicated in Supplementary Table S10.

### Statistics

All experiments were performed at least in biological triplicates. Statistical significance was tested by a parametric Student's *t*-test on equally distributed data. Otherwise, a non-parametric Mann–Whitney-test was performed. For Kaplan–Meier analyses, statistical significance was determined by log-rank analyses.

## RESULTS

### IGF2BP1 is a conserved enhancer of tumor cell proliferation

**Figure 1.**
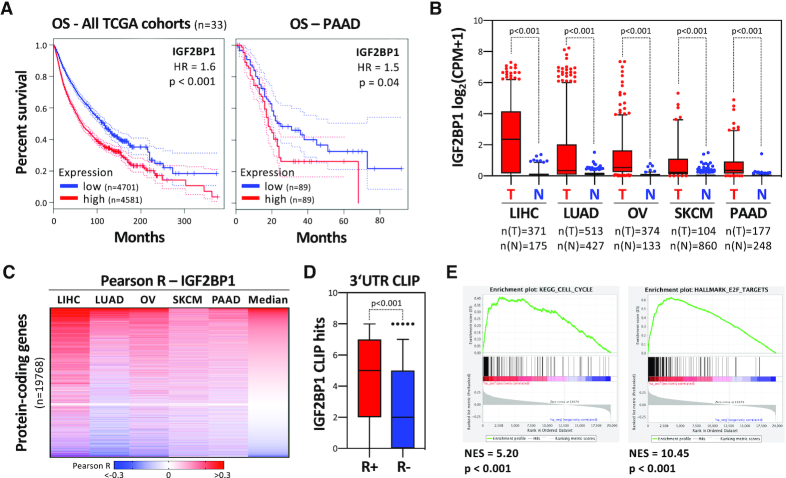
IGF2BP1 is a conserved pro-oncogenic RBP in human cancer. (**A**) Kaplan–Meier plots of overall survival analyses (median cutoff) based on IGF2BP1 mRNA expression. Overall survival was analyzed for all TCGA (The Cancer Genome Atlas) tumor cohorts (9282 patients, left) and the PAAD cohort (Pancreatic Adenocarcinoma, 178 patients, right). Red, high expression of IGF2BP1; Blue, low expression of IGF2BP1. HR, hazard ratio; p, logrank p value. (**B**) Box plots showing the IGF2BP1 expression in tumor and normal tissues for indicated cancers. Data were derived from the TCGA (T, red boxes) and GTEx (N, blue boxes) portal. The number (*n*) of analyzed samples is indicated. LIHC, liver hepatocellular carcinoma; LUAD, lung adenocarcinoma; OV, ovarian carcinoma; SKCM, skin cutaneous melanoma; PAAD, pancreatic adenocarcinoma). (**C**) Heatmap of correlation coefficients (R) determined for protein-coding gene and IGF2BP1 expression. *R* values were determined in indicated TCGA data sets and ranked according to median correlation coefficient. Scale bar in lower panel. (**D**) Box plots of experiments indicating IGF2BP1 CLIP hits in the 3′UTR of mRNAs showing positive (R+; *R* > 0.15, *n* = 2039) or negative (*R–*; *R*←0.15, *n* = 155) association with IGF2BP1 expression in cancers analyzed in (C). (**E**) Gene set enrichment analysis (GSEA) of IGF2BP1-correlated gene expression in the five cancers analyzed in (C). GSEA was performed on ranked median correlation coefficient determined in (C). KEGG pathway ‘Cell Cycle’ (left) and the Hallmark pathway ‘E2F Targets’ (right) are shown. NES, normalized enrichment score. Statistical significance was determined by Mann–Whitney test.

The IGF2 mRNA-binding protein 1 (IGF2BP1) promotes the proliferation and *in vivo* growth of tumor cells derived from a variety of solid cancers (8,9,32). In agreement, the meta-analysis of 33 TCGA-provided cancer transcriptome data sets, including 9282 tumor samples, indicated that high IGF2BP1 expression is associated with reduced overall survival probability (Figure 1A). For 9 out of 33 cancers, high IGF2BP1 mRNA expression was significantly (*P* < 0.05) associated with adverse prognosis (Supplementary Figure S1A). This included pancreatic adenocarcinoma (Figure 1A; PAAD). In the vast majority of cancers, IGF2BP1 synthesis was substantially upregulated, supporting its oncofetal expression and conserved prognostic relevance. Among cancers with substantially upregulated IGF2BP1 expression were four with reported pro-oncogenic roles of IGF2BP1 (LIHC, LUAD, OV and SKCM) as well as PAAD (Figure 1B). In these five cancers, IGF2BP1 followed by LIN28B were the in average most upregulated mRNA-binding proteins (mRBPs) among 660 detected and reported by the RBP census (Supplementary Figure S1B) (33). IGF2BP1 is a known regulator of mRNA stability and associated with a plethora of mRNAs. CLIP studies in different cell types suggested thousands of IGF2BP1-bound mRNAs. So far, many studies focused on the IGF2BP1-dependent stabilization of the MYC mRNA. Surprising in view of the reported MYC/N-enhancing role of IGF2BPs (10,12,34), no significant association of IGF2BP1 and MYC was observed across 33 cancers or the five cancers (LIHC, LUAD, OV, SKCM and PAAD) with expected or validated pro-oncogenic roles of IGF2BP1 (Supplementary Figure S1C, D). This suggested that IGF2BP1 acts via largely cancer-specific pathways or that its most conserved effector pathway(s) across cancer types remained unknown.

Aiming to identify key candidate effector pathways, IGF2BP1-associated expression of protein-coding genes was investigated in the aforementioned five cancers (Supplementary Table S1). The median correlation coefficient was used to rank genes and distinguish two major groups, genes showing positive (R+) or negative (R–) correlation with IGF2BP1 expression (Figure 1C). The investigation of IGF2BP1–3′UTR association, re-analyzed in eight independent CLIP studies performed in four distinct cell types (8), suggested an enrichment of conserved 3′UTR-binding among the positively correlated (R+) transcripts (Figure 1D). This supported IGF2BP1’s role as a mainly 3′UTR-dependent mRNA-stabilizing mRBP in cancer. Gene set enrichment analyses (GSEA) of genes ranked by their determined median association with IGF2BP1 expression in the five investigated solid cancers demonstrated a striking enrichment of R+-genes in the E2F_TARGETS hallmark as well as the KEGG_CELL_CYCLE gene sets (Figure 1E; Supplementary Table S2). This significant enrichment was also observed in each of the five investigated cancers suggesting E2F-driven gene expression as a conserved effector pathway of IGF2BP1 in cancer (Supplementary Figure S2A–C; Table S2).

### IGF2BP1 promotes cell cycle progression in cancer-derived cells

**Figure 2.**
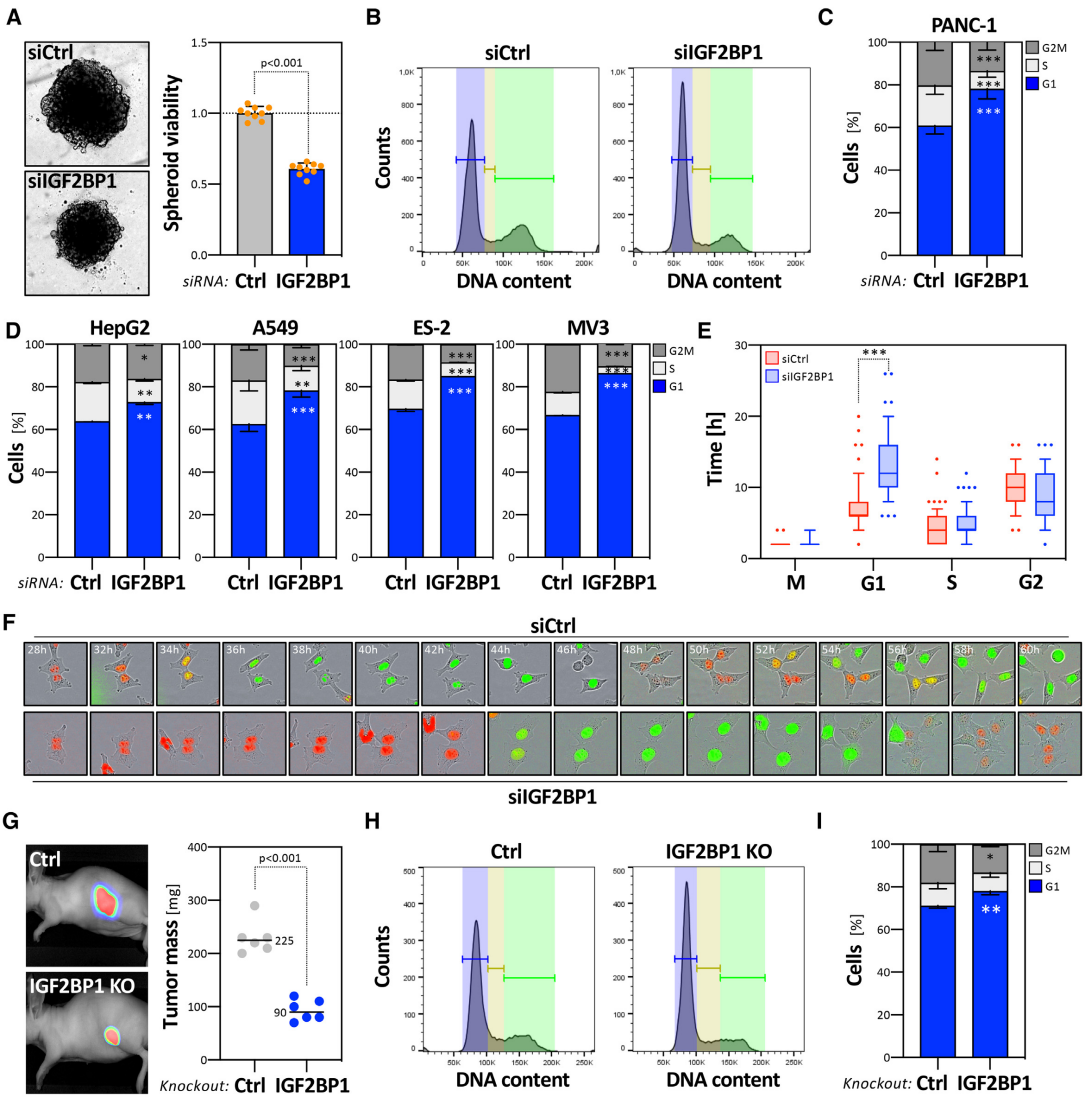
IGF2BP1 promotes proliferation and cell cycle progression in cancer cells. (**A**) PANC-1 cells were transfected with control (siCtrl, gray) or IGF2BP1-directed siRNA pools (siIGF2BP1, blue). Representative PANC-1 spheroids 6 days post-transfection are indicated in the left panel. The viability of PANC-1 spheroids was determined by CellTiter GLO (right panel). Orange dots represent median-normalized values of three spheroids analyzed in three independent studies. (B–D) PANC-1 cell cycle phase distribution upon transfection with control (**B**, left panel) or IGF2BP1-directed (**B**, right panel) siRNAs, as determined by PI-labeling and flow cytometry. Fractions of PANC-1 (**C**) or HepG2, A549, ES-2 and MV3 (**D**) cells in each cell cycle phase were quantified in three independent knockdown analyses. (**E**) Box plots showing the duration of cell cycle phases upon control (siCtrl, red) or IGF2BP1-depletion (siIGF2BP1, blue). ES-2 cells were stably transduced with the FUCCI system (Sartorius). The length of cell cycle phases was determined over 66 (control, red) and 80 (IGF2BP1-depleted, blue) cell divisions. (**F**) Representative images of cells with segmentation mask overlays analyzed in (E) at indicated time post transfection with control- (upper panel) or IGF2BP1-directed (bottom panel) siRNAs. Red, G1 phase; Green, G2 phase; Yellow, S phase. (**G**) Parental (Ctrl) and IGF2BP1-deleted (KO) A549 cells expressing iRFP were injected (sc) into nude mice (6 mice per condition) and the growth of xenograft tumors was monitored by near-infrared imaging. Representative images are shown in the left panel (42 days post-injection). Final tumor mass is shown by box plots (right panel). (**H, I**) Cell cycle analysis, as presented in (B, C) of parental and IGF2BP1-deleted A549 cells. Statistical significance was determined by Mann–Whitney test: *P <0.05; **P < 0.01; ***P < 0.001.

If and how IGF2BP1 controls E2F-dependent cell cycle control and proliferation was initially investigated in PAAD-derived PANC-1 cells. IGF2BP1 depletion impaired spheroid growth, significantly decreased 2D cell proliferation and elevated doubling time approximately twofold (Figure 2A; Supplementary Figure S3A, B). Cell cycle progression analyses showed that IGF2BP1 knockdown led to an enrichment of cells in G1 (Figure 2B, C). Importantly, IGF2BP1 depletion was not associated with increased apoptosis, as indicated by barely observed subG1 cell fractions or dead cells, monitored by PI-labeling (Supplementary Figure S3C). IGF2BP1 knockdown also impaired colony formation and clonogenicity suggesting a pivotal role of cell cycle control and sustained self-renewal potential (Supplementary Figure S3D, E). Impaired G1/S-progression upon IGF2BP1 depletion was also observed in four cell lines derived from the four other cancers investigated here (Figure 2D; Supplementary Figure S4A). The monitoring of cell cycle progression at the single cell level (65 divisions) in IGF2BP1-depleted ES-2 cells (OV-derived), using the FUCCI technology (35), demonstrated that IGF2BP1 depletion exclusively prolonged the G1 phase, approximately twofold (Figure 2E, F). Population analyses over 72 h including 4800 cells confirmed this by indicating a substantial increase of cells in G1 and decrease in the S phase upon knockdown (Supplementary Figure S4B, C). This was further evaluated by IGF2BP1 deletion (KO) in LUAD-derived A549 cells. IGF2BP1-KO impaired spheroid growth (Supplementary Figure S4D), as previously observed in other cancer-derived cells (8). Furthermore, spheroid growth was significantly enhanced in A549 knockout cells by the re-expression of wild type (WT) GFP-tagged IGF2BP1 (Supplementary Figure S4E). Compared to GFP re-expressing controls, viability remained essentially unchanged by the re-expression of an RNA-binding deficient mutant GFP-IGF2BP1 (MUT). This indicated the importance of the IGF2BP1’s RNA-binding capacity in controlling tumor cell proliferation and spheroid growth. In nude mice, IGF2BP1 deletion substantially interfered with the growth of A549-derived xenograft tumors (Figure 2G). Concise with its depletion, IGF2BP1 loss resulted in an increase of A549 cells in G1 without enhanced subG1 cell fractions (Figure 2H, I). This indicated, IGF2BP1 is a conserved regulator of G1/S-transition in cancer cells, controlling tumor cell proliferation *in vitro* and tumor growth *in vivo*.

### IGF2BP1 regulates the E2F-dependent control of G1/S-transition

**Figure 3.**
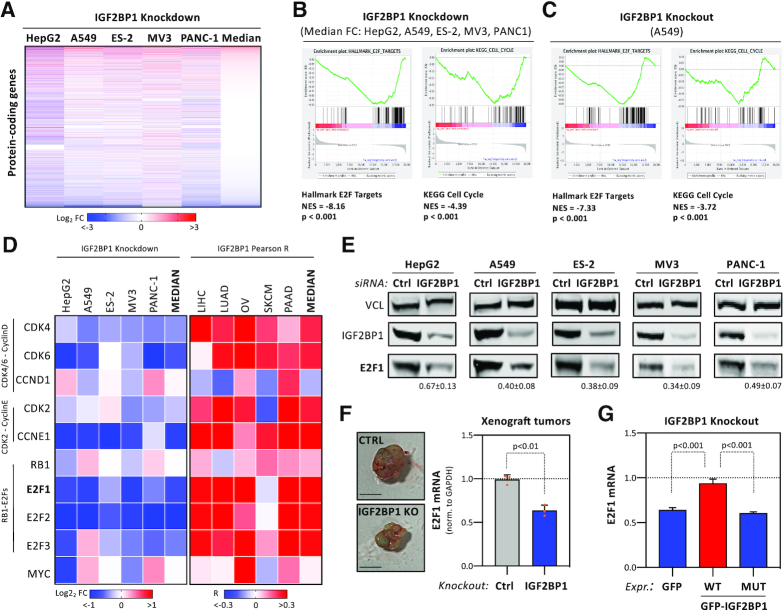
IGF2BP1 controls G1/S cell cycle transition of cancer cells. (**A**) Heatmap indicating the fold change (FC) of mRNAs upon IGF2BP1-depletion in indicated cancer cell lines 72 h post-transfection of siRNAs. The abundance of mRNAs was monitored by RNA sequencing. Genes were ranked according to their median FC determined in five cancer cell lines. (B, C) Gene set enrichment analysis (GSEA) of median FC of protein-coding genes upon IGF2BP1 depletion in five cancer cell lines (**B**) or upon IGF2BP1 knockout in A549 cells (**C**). Results for the KEGG pathway ‘Cell Cycle’ (left) and the Hallmark pathway ‘E2F Targets’ (right) are shown. NES, normalized enrichment score. (**D**) Heatmap depicting the fold change of indicated mRNAs upon IGF2BP1 depletion in cancer cells (left) and their correlation (coefficient) with IGF2BP1 mRNA expression in indicated cancers (right). Median fold change or correlation coefficients are indicated in most right columns. (**E**) Representative Western blot analyses of E2F1 upon IGF2BP1 depletion in indicated cancer cell lines. Vinculin (VCL) served as a loading and normalization control. Average fold change and standard deviation of E2F1 protein levels, determined in three independent analyses are indicated in bottom panel. (**F**) RT-q-PCR analysis of E2F1 mRNA levels in excised xenograft tumors (Figure 2G). Representative images of tumors are shown in the left panel. E2F1 expression was determined in three control and IGF2BP1-KO tumors by normalization to GAPDH. (**G**) RT-q-PCR analysis of E2F1 mRNA levels in IGF2BP1-KO A549 cells expressing GFP, GFP-IGF2BP1 (WT) or an RNA-binding deficient GFP-IGF2BP1 (MUT) normalized to parental A549 cells. RPLP0 served as normalization control. Statistical significance was determined by Student's t-test.

To identify conserved key effector-encoding mRNAs of IGF2BP1-dependent control of G1/S-transition, the expression of protein-coding genes was monitored upon IGF2BP1 depletion by RNA-seq in five tumor cell lines (Supplementary Table S3). For GSEA, protein-coding genes were ranked by their median or cell-specific fold change of expression (Figure 3A). This indicated conserved and significant downregulation of the E2F_TARGET and KEGG cell cycle gene sets in median and all individual cell models, including IGF2BP1-KO A549 cells (Figure 3B, C; Supplementary Figure S5A; Supplementary Table S4). In agreement, IGF2BP1 depletion resulted in the largely conserved downregulation of factors promoting G1/S transition (Figure 3D, left panel; Supplementary Table S5). These genes also showed significant and concise association with IGF2BP1 expression in the five respective primary cancers (Figure 3D, right panel). No evidence for IGF2BP1-dependent regulation of factors impairing G1/S transition, e.g. RB1, was observed. Surprisingly, significant downregulation of MYC mRNA abundance was only determined in HepG2 and MV3 cells, supporting previous studies (9,12). In view of lacking correlation of IGF2BP1 and MYC mRNAs across cancers (see Supplementary Figure S1C and S1D), this provided further evidence that IGF2BP1-dependent regulation of MYC mRNA levels is rather cell-type specific. In contrast, these findings indicated a pivotal role of IGF2BP1 in regulating E2F-driven transcription in a concise manner across different cancers and tumor cell lines. This was tested further for the E2F1–3 transcription factors, well known enhancers of G1/S transition (17). In all analyzed cancer cells, IGF2BP1 depletion significantly reduced E2F1–3 mRNA levels, with the exception of A549 cells where E2F3 transcripts were modestly elevated (Figure 3D). In agreement with decreased mRNA abundance, E2F1 protein expression was substantially reduced in all tested cancer cell lines upon IGF2BP1 depletion (Figure 3E). Likewise, E2F2 and E2F3 protein levels were decreased by IGF2BP1 knockdown in PANC-1 cells (Supplementary Figure S5C). Notably, downregulation of E2F1 protein was also observed in A549 IGF2BP1-KO cells (Supplementary Figure S5D). Furthermore, E2F1 mRNA levels were strongly reduced in xenograft tumors lacking IGF2BP1 (Figure 3F). In GFP-expressing A549 cells deleted for IGF2BP1, E2F1 mRNA and protein levels were reduced nearly twofold compared to parental cells (Figure 3G, GFP; Supplementary Figure S5E). The re-expression of wild type IGF2BP1 restored E2F1 mRNA and protein expression (Figure 3G, WT; Supplementary Figure S5E). This was not observed upon the re-expression of RNA-binding deficient IGF2BP1, suggesting that IGF2BP1 controls E2F1 expression in an RNA-binding dependent manner (Figure 3G, MUT; Supplementary Figure S5E (8)).

### IGF2BP1 regulates E2F1 expression in a 3′UTR- and miRNA-dependent manner

**Figure 4.**
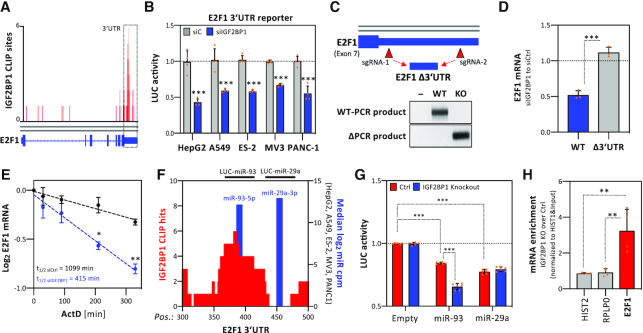
IGF2BP1 enhances E2F1 mRNA stability in a 3′UTR- and miRNA-dependent manner. (**A**) IGF2BP1 CLIP profile of the E2F1 mRNA. The 3′UTR is highlighted by dashed lines. (**B**) E2F1 3′UTR luciferase activity in indicated cancer cells upon control- or IGF2BP1-depletion. Reporter activities, normalized to a control reporter, were determined in four independent experiments. (**C**) Experimental strategy of the genomic deletion of the E2F1 3′UTR by the Cas9 nuclease using indicated CRISPR guide RNAs (sgRNAs) (Top panel). Representative PCR analysis on genomic DNA of parental PANC-1 (WT) and an E2F1 3′UTR-deleted PANC-1 cell clone (KO). (**D**) RT-q-PCR analysis of E2F1 mRNA levels upon IGF2BP1 depletion in parental PANC-1 (WT, blue) or the E2F1 3′UTR-deleted PANC-1 cell clone (Δ3′UTR, grey) normalized to control-transfected cells. RPLP0 served as internal normalization control. (**E**) E2F1 mRNA decay was monitored by RT-q-PCR in control- (black) or IGF2BP1-depleted (blue) PANC-1 cells upon indicated time of Actinomycin D (ActD) treatment. Error bars indicated standard deviation. Average mRNA half-life, determined in three independent studies, is indicated. (**F**) The graph depicts the number of CLIP studies showing overlapping IGF2BP1-CLIP sites (left axis, red; CLIP hits) and the position (x-axis) of miRNA targeting sites (blue) in the E2F1 3′UTR. MiRNA abundance (right axis, blue) is indicated as median log_2_ cpm determined by small RNA-seq of indicated cell lines. Luciferase reporter comprising indicated regions of the E2F1–3′UTR (LUC-miR-93, LUC-miR-29a) and analyzed in (G), are indicated in the top panel. (**G**) Luciferase reporter analysis demonstrating activity of indicated reporters in parental (Ctrl, red) or IGF2BP1-knockout (blue) A549 cells. Reporter activities, normalized to a control reporter without miRNA targeting site (Empty), were determined in three independent experiments with two technical replicates each. (**H**) Co-purification of mRNAs with AGO2 in parental (Ctrl) or IGF2BP1-knockout A549 cells was analyzed by immunoprecipitation using anti-AGO2 antibodies and RT-q-PCR analysis (right panel). HIST2 and RPLP0 served as negative controls. HIST1 served as normalization control. Statistical significance was determined by Student's *t*-test: *P <0.05; **P < 0.01; ***P < 0.001.

IGF2BP1’s main and conserved role in cancer-derived cells relies on the 3′UTR- and miRNA-dependent regulation of mRNA turnover (8). IGF2BP1-mRNA association reported by CLIP indicated conserved binding to the 3′UTR of the E2F1 mRNA (Figure 4A). Preferred 3′UTR-binding was also observed for other transcripts encoding positive regulators of G1/S transition (Supplementary Figure S5B). 3′UTR-dependent regulation was tested for E2F1 using E2F1–3′UTR containing luciferase reporters. IGF2BP1 depletion resulted in conserved downregulation of reporter activity in all analyzed cancer cell lines (Figure 4B). This was further investigated by deleting the E2F1–3′UTR in PANC-1 cells by sgRNAs directing Cas9-cleavage 3′-to the stop codon and 5′-to the polyadenylation signal (Figure 4C). IGF2BP1 expression remained unaffected by homozygous deletion of the bulk E2F1–3′UTR. However, 3′UTR-deletion abolished downregulation of E2F1 mRNA levels observed upon IGF2BP1 knockdown in parental PANC-1 cells (Figure 4D). These findings strongly suggested that IGF2BP1 controls E2F1 mRNA turnover via the 3′UTR. In accord, IGF2BP1 knockdown significantly enhanced decay of the E2F1 mRNA upon blocking transcription by actinomycin D (Figure 4E; ActD). Notably, although IGF2BP1 depletion reduced total E2F1 mRNA levels, the synthesis of nascent E2F1 mRNAs remained essentially unchanged (Supplementary Figure S6A). Together, this indicated that IGF2BP1 exclusively controls E2F1 mRNA turnover without substantial deregulation of E2F1 mRNA synthesis.

E2F1 expression is controlled at various levels including miRNA-directed inhibition via the 3′UTR (17,19). To identify conserved miRNAs controlling E2F1 synthesis, the expression of miRNAs was monitored in the five investigated cell lines (Supplementary Table S6). MiRNA abundance was then plotted over the number of databases, analyzed via MiRWalk2.0, predicting miRNA targeting at the E2F1–3′UTR (Supplementary Figure S6B). These studies revealed high and conserved abundance of various miRNAs with *in silico* predicted or validated E2F1 repression, e.g. miR-29a-3p and miR-93–5p (19). IGF2BP1 binding sites in the E2F1 3′UTR reported by CLIP studies overlap with the predicted seed region of the miR-93–5p family (Figure 4F). In contrast, no CLIP-reported binding was observed in the predicted miR-29a-3p seed. To test if IGF2BP1 modulates regulation by these miRNAs via predicted targeting sites, we analyzed luciferase reporters containing 48 nucleotide long E2F1 3′UTR regions including the aforementioned miRNA seeds (Figure 4G). Luciferase activity for both reporters was reduced in A549 cells compared to a reporter encoding a minimal 3′UTR suggesting regulation by miRNAs, as predicted. However, in A549 cells deleted for IGF2BP1, activity of the miR-93-5p reporter was significantly decreased compared to parental cells. In contrast, activity of the miR-29a-3p reporter remained unaffected by IGF2BP1 deletion, suggesting that IGF2BP1 impairs miR-93–5p directed regulation by impairing miRNA regulation due to binding at or in proximity to the miR targeting site. To exclude bias by IGF2BP1-KO, this was further investigated by analyzing miR-93-5p reporter activity upon IGF2BP1 knockdown (Supplementary Figure S6C). As observed in IGF2BP1-KO cells, reporter activity was also decreased upon IGF2BP1 depletion. Finally, we analyzed AGO2-association of the E2F1 mRNA by RIP in A549 cells. In comparison to parental cells, AGO2-association of control mRNAs remained unaffected by IGF2BP1 deletion (Figure 4H, Supplementary Figure S6D). In sharp contrast, AGO2-E2F1 mRNA association was increased in IGF2BP1-KO cells, indicating that IGF2BP1 impairs miRNA-directed downregulation of E2F1 expression.

### The E2F pathway is controlled by IGF2BP1 in an m^6^A-dependent manner

**Figure 5.**
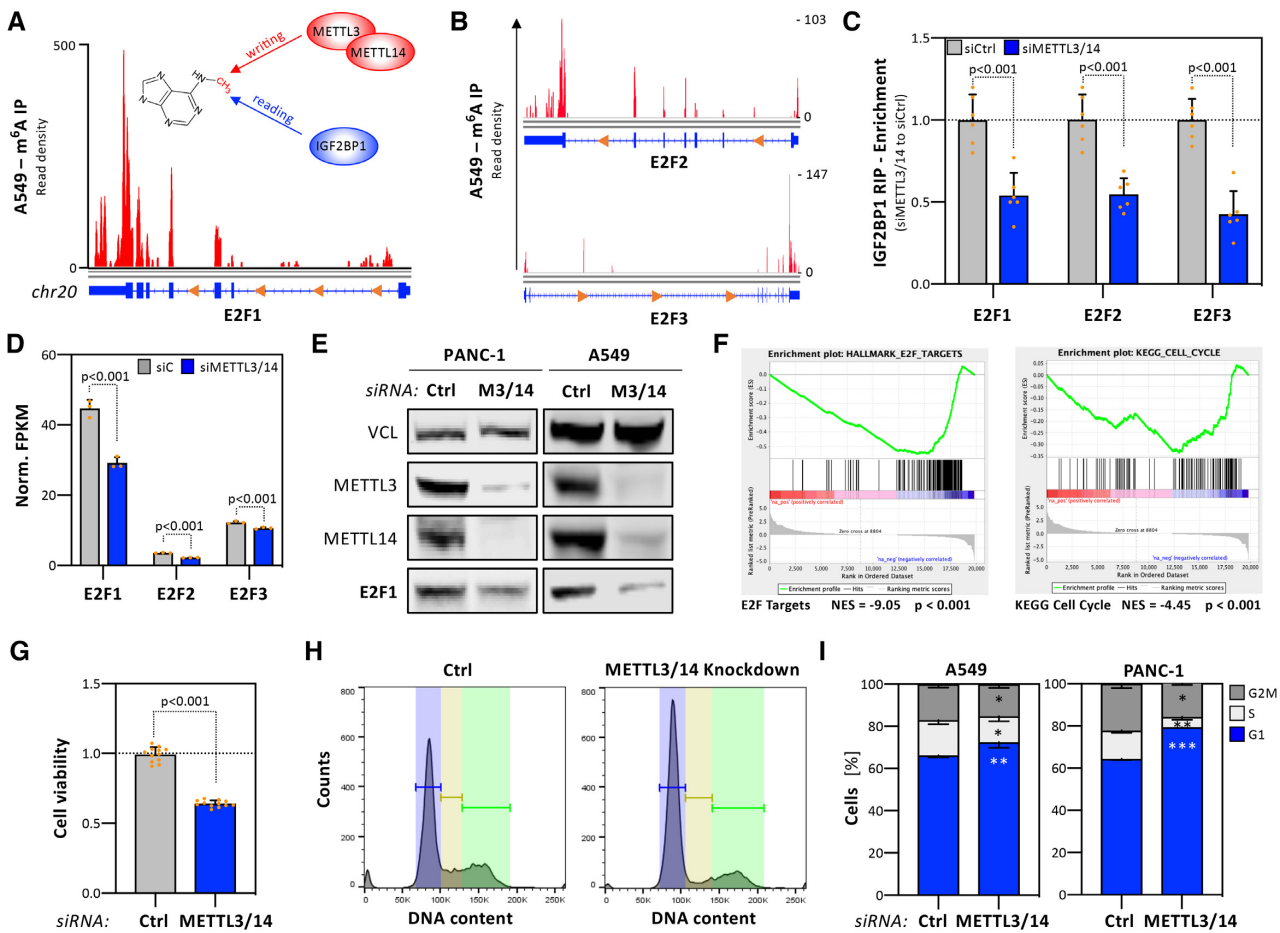
The E2F-dependent cell cycle progression controlled by IGF2BP1 is m^6^A-dependent. (A, B) N^6^-Methyladenosine RIP-seq profile of the E2F1 (**A**), E2F2 and E2F3 (**B**) mRNAs determined in A549 cells. Data were obtained from MeT-DB V2.0. The m^6^A-writing enzymes METTL3 and METTL14 as well as IGF2BP1 as an m^6^A-reader are indicated. (**C**) Co-purification of indicated mRNAs with IGF2BP1 in control- (gray, siCtrl) or METTL3/14-depleted (blue, siMETTL3/14) PANC-1 cells was analyzed by RIP using anti-IGF2BP1 antibodies and RT-q-PCR analysis. HIST1 served as normalization control. (**D**) Abundance of E2F1–3 mRNAs determined by RNA-seq upon transfection of indicated siRNAs in PANC-1 cells as shown in (**C**). (**E**) Representative Western blot analysis of indicated proteins upon control- and METTL3/14 depletion in PANC-1 (left) and A549 (right) cells. VCL served as loading control. (**F**) Gene set enrichment analysis (GSEA) of protein-coding genes upon METTL3/14 co-depletion in PANC-1 cells. Protein-coding genes were ranked by their fold change of mRNA abundance determined by RNA-seq as shown in (**D**). Results for the Hallmark pathway ‘E2F Targets’ (left) and the KEGG pathway ‘Cell Cycle’ (right) are shown. NES, normalized enrichment score. (**G**) PANC-1 cells were transfected with indicated siRNAs (grey, siCtrl; siMETTL3/14, blue). The viability of PANC-1 spheroids was determined 6 days post-transfection by CellTiter GLO, as described in Figure 2A. (**H, I**) Cell cycle progression analyses of control- or METTL4/14-depleted PANC-1 and A549 cells (**I**), as described in Figure 2B, C. Representative cell cycle phase distribution in PANC-1 cells is shown in (H). Statistical significance was determined by Mann–Whitney test: *P < 0.05; **P < 0.01; ***P < 0.001.

IGF2BPs are major m^6^A-readers in cancer, showing an m^6^A-dependent increase in target mRNA association (12,16). In A549 and other cells, m^6^A-RIP studies indicated preferential m^6^A-modification of E2F1–3 mRNAs in the 3′UTR close to the stop codon (Figure 5A, B). All these mRNAs were consistently decreased by IGF2BP1 depletion and show conserved 3′UTR-association in IGF2BP1-CLIP studies (Figure 3D, 4A; Supplementary Figure S5B). In PANC-1 cells, IGF2BP1-RIP studies demonstrated that co-depletion of METTL3/14, crucial for m^6^A-modification of mRNAs, significantly reduced IGF2BP1-association of all three mRNAs (Figure 5C). In PANC-1 cells, METTL3/14 co-depletion resulted in reduced E2F1–3 mRNA and protein levels (Figure 5D, E; Supplementary Figure S6E and Table S7). The co-depletion of METTL3/14 and METTL3-deletion by CRISPR/Cas9 in A549 cells led to reduced E2F1 expression without affecting IGF2BP1 abundance (Figure 5E; Supplementary Figure S6F). GSEA of gene expression determined by RNA-seq upon METTL3/14 co-depletion confirmed significant downregulation of E2F_ target and KEGG cell cycle gene sets (Figure 5F; Supplementary Table S8). This was associated with impaired cell viability and cell cycle progression, evidenced by accumulation of cells in G1 (Figure 5G–I). Furthermore, the expression of IGF2BP1, METTL3 and METTL14 is strongly correlated with E2F1–3 expression (*R* = 0.4) across 33 TCGA tumor cohorts (Supplementary Figure S6G). In sum, this indicated that IGF2BP1 promotes E2F-driven G1/S transition in an m^6^A-dependent manner.

### IGF2BP1 is a post-transcriptional super-enhancer of E2F-dependent transcription

**Figure 6.**
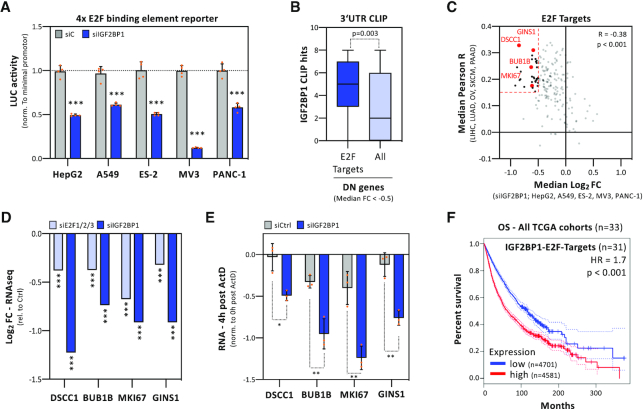
IGF2BP1 is a post-transcriptional enhancer of E2F-driven genes. (**A**) E2F-responsive promoter studies. Luciferase activities, normalized to minimal promoter activity, were determined in indicated cancer cells upon control- (gray) or IGF2BP1-depletion (blue) in four independent experiments. (**B**) Box plots of IGF2BP1 CLIP hits in the 3′UTR of mRNAs showing a median log_2_FC < –0.5 upon IGF2BP1 depletion, as determined in five cancer cell lines (see Figure 3A). E2F Targets, *n* = 196; All, *n* = 1280. (**C**) The median correlation coefficient (*R*) of E2F target genes with IGF2BP1 in indicated primary cancers was plotted against the median log_2_FC observed upon IGF2BP1 depletion in indicated cancer cells. Dashed lines distinguish genes with *R* > 0.15 and log_2_FC←0.5 (*n* = 31). (**D**) Log_2_ FC of DSCC1, BUB1B, MKI67 and GINS1 upon E2F1/2/3- or IGF2BP1-depletion in PANC-1 cells, as determined by RNA seq. (**E**) mRNA decay of indicated genes was monitored by RT-q-PCR in control- (gray) or IGF2BP1-depleted (blue) PANC-1 cells upon 4h of Actinomycin D (ActD) treatment and normalized to RNA levels prior ActD treatment. Error bars indicated standard deviation in three independent studies. RPLP0 served as internal normalization control. (**F**) Kaplan–Meier plots of overall survival analyses (median cutoff) based on the expression of 31 IGF2BP1 and E2F target mRNA (as shown in C) expression. Overall survival was analyzed for all 33 TCGA tumor cohorts (9282 patients). HR, hazard ratio; *P*, logrank *P* value. Statistical significance was determined by Mann–Whitney test: *P < 0.05; **P < 0.01; ***P < 0.001.

Our studies suggested that IGF2BP1 is a conserved post-transcriptional enhancer of E2F-driven transcription in cancer. In agreement, the activity of E2F-promoter luciferase reporters was consistently decreased by IGF2BP1 depletion in all here investigated cancer cell lines (Figure 6A). The evaluation of IGF2BP1-CLIP studies revealed enriched 3′UTR-association of E2F_TARGET transcripts among mRNAs downregulated by IGF2BP1 depletion in five cancer cell lines (Figure 6B, dark to light blue). Moreover, E2F_TARGET genes showed a strong correlation of downregulation upon IGF2BP1 depletion in cancer cell lines and IGF2BP1-associated expression in the corresponding primary cancers (Figure 6C). This suggested that IGF2BP1 promotes the synthesis of E2F-driven gene expression via E2Fs and stabilizes the respective mRNAs. This was analyzed for four E2F-driven transcripts: DSCC1, BUB1B, MKI67 and GINS1 (Figure 6C, red). These were among 31 transcripts (black and red) showing association with IGF2BP1 in primary tumors (R > 0.15) and consistent downregulation (log_2_FC < –0.5) upon IGF2BP1 depletion in cancer cells (Figure 6C, dashed lines). IGF2BP1-CLIP indicated conserved and 3′UTR-directed association for all four mRNAs (Supplementary Figure S7A). Notably, one transcript, the proliferation marker Ki-67 (MKI67), is stabilized by IGF2BP1 in hepatocellular carcinoma (9). RNA-seq confirmed that all four mRNAs were downregulated by IGF2BP1 knockdown and the co-depletion of E2F1–3 (Figure 6D; Supplementary Table S9). Co-depletion of E2F1–3 resulted in severely impaired 2D proliferation and spheroid growth, as expected (Supplementary Figure S7B, C). The analysis of mRNA turnover upon blocking transcription indicated significant destabilization of all four transcripts upon IGF2BP1 knockdown (Figure 6E). Like observed for IGF2BP1 (see Figure 1A), the mRNA expression of the 31 identified E2F/IGF2BP1-driven factors was associated with a significantly reduced survival probability across 33 cancers (Figure 6F). Moreover, these genes showed conserved association with IGF2BP1 expression across these 33 cancers as well as the aforementioned five cancers primarily investigated here (Supplementary Figure S7D). In conclusion, this indicated that IGF2BP1 is a post-transcriptional ‘super’-enhancer of E2F-driven gene expression. The protein promotes the E2F hallmark pathway by enhancing E2F1–3 abundance and stabilizes E2F-driven oncogenic transcripts.

### BTYNB inhibits enhancement of E2F-driven gene expression by IGF2BP1

**Figure 7.**
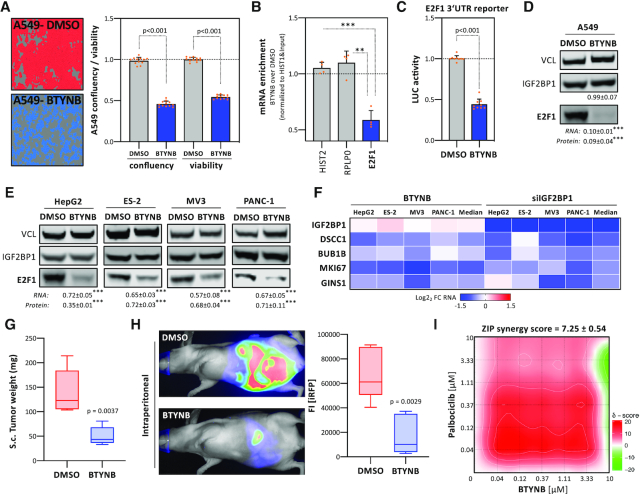
BTYNB impairs IGF2BP1’s post-transcriptional super-enhancer function in E2F-driven gene expression. (**A**) A549 cells were treated with DMSO (red) or 5 μM BTYNB (blue) for 48 h. Representative images are indicated in the left panel. Cell confluency and viability were determined by an IncuCyte S3 analyzer and CellTiter GLO (right panel). (**B**) Co-purification of mRNAs with IGF2BP1 in A549 cells upon treatment with 5 μM BTYNB (48 h) was analyzed by immunoprecipitation using anti-IGF2BP1 antibodies and RT-q-PCR analysis. HIST2 and RPLP0 served as negative controls. HIST1 served as normalization control. (**C**) E2F1 3′UTR luciferase activity in A549 cells upon DMSO- or BTYNB (5 μM) treatment. Reporter activities were determined in three independent experiments, including three technical replicates each, as described in Figure 4B. (**D, E**) Western blot analysis of E2F1 protein level upon DMSO or BTYNB (5 μM, 48 h) treatment in indicated cancer cells. The fold change of E2F1 protein and mRNA levels, determined by RT-q-PCR, upon BTYNB treatment is indicated in bottom panel. (**F**) Heatmap showing log_2_ FC of indicated mRNAs upon 5 μM BTYNB treatment (left) or IGF2BP1 depletion (right) in indicated cancer cells. (G, H) iRFP-labeled ES-2 cells were treated with DMSO or 5 μM BTYNB for 24h and injected sc (**G**) or ip (**H**) into nude mice (5 mice per condition). The mass of final sc tumors (G) is shown by box plots. Representative images indicating iRFP-labeled intraperitoneal tumors are shown in the left panel (H). The total, final iRFP fluorescence intensity (FI) of ip tumor burden is indicated by box plots (H, right panel). (**I**) Relief plot showing the ZIP synergy for combined treatment with BTYNB and Palbociclib (72 h) at indicated concentrations in 2D-cultured ES-2 cells. Cell viability was determined using Cell Titer GLO. Synergy maps were generated using the SynergyFinder web application (https://synergyfinder.fimm.fi, (31)). ZIP (zero interaction potency) synergy scores were determined in four independent experiments. Synergy scores are color-coded (scores < –10, antagonistic, green; scores > 10, synergistic, red). Statistical significance was determined by Mann–Whitney test: *P <0.05; **P < 0.01; ***P < 0.001.

Previous studies reported a small molecule inhibitor, BTYNB (21), impairing association of IGF2BP1 with the MYC RNA *in vitro*. BTYNB interfered with cancer cell proliferation and expression of some prior known IGF2BP1 target transcripts. This suggested that this lead compound may as well disrupt E2F/IGF2BP1-driven gene expression. BTYNB exposure (48 h) impaired the viability of LUAD-derived A549 cells (Figure 7A). IGF2BP1-RIP analyses demonstrated that BTYNB treatment for 24 h, when cell vitality was barely affected (data not shown), is associated with reduced binding of IGF2BP1 to the E2F1 mRNA (Figure 7B; Supplementary Figure S8B). In contrast, IGF2BP1-association with the E2F1 mRNA remained essentially unaffected by treatment (24 h) with Palbociclib (36), a CDK4/6-targeting cell cycle inhibitor currently in phase 4 clinical trials (Supplementary Figure S8B, C). These findings suggested that BTYNB impairs IGF2BP1-association with the E2F1 mRNA. Consistently, BTYNB also led to significantly reduced activity of E2F1–3′UTR luciferase reporters and decreased E2F1 expression at the protein as well as mRNA level (Figure 7C, D). Likewise, BTYNB decreased E2F1 expression and vitality of all other here investigated cancer cell lines without affecting IGF2BP1 abundance (Figure 7E; Supplementary Figure S8A). If BTYNB also impacts the expression of prior identified E2F1-driven target transcripts of IGF2BP1 (DSCC1, BUB1B, MKI67 and GINS1) was analyzed by IGF2BP1-RIP and monitoring steady state mRNA levels in A549 cells. In agreement with reduced IGF2BP1-association of the four mRNAs, steady state levels of all four transcripts were markedly reduced upon BTYNB exposure of A549 as well as all other cancer cell lines investigated (Figure 7F; Supplementary Figure S8D, E).

How BTYNB treatment impairs tumor initiation and growth was analyzed in iRFP-labeled (near infrared red fluorescent protein) ES-2 cells. In these, the deletion of IGF2BP1 impaired the growth of subcutaneous (s.c.) xenograft tumors and interfered with metastasis (8). ES-2 cells exposed to BTYNB, prior (24 h) and during s.c. injection of viable tumor cells, formed tumors at the same efficiency observed for DMSO-treated controls (Figure 7G; Supplementary Figure S8F). However, already 7 days post s.c. injection tumor growth was markedly reduced by BTYNB. This was observed up to 3 weeks post last BTYNB treatment. A major problem of ovarian cancer progression is the rapid spread of malignancies in the peritoneum. If BTYNB also interferes with the peritoneal growth of ES-2 cells was monitored upon intraperitoneal (i.p.) injection. BTYNB treatment impaired both, the growth and spread of tumor cells with reduced tumor burden observed up to 2 weeks after the last treatment (Figure 7H; Supplementary Figure S8G).

Our studies implied that the indirect, IGF2BP1-directed impairment of E2F-driven gene expression by BTYNB synergizes with other cell cycle inhibitors like Palbociclib, targeting activating kinases upstream of E2Fs. This was tested by matrix analysis of combinatorial treatment in ES-2 cells. These analyses revealed synergy scores larger than 7, indicating additive effects (Synergy score between –10 and 10) of BTYNB and Palbociclib according to the ZIP (zero interaction potency) synergy model (Figure 7I). Notably additivity or even synergy was observed already at low concentrations of both compounds, providing promising evidence that the inhibition of IGF2BP1-RNA association by BTYNB is beneficial in combined treatment aiming to impair tumor cell proliferation.

## DISCUSSION

Our studies reveal that IGF2BP1 is the first RBP acting as a conserved post-transcriptional enhancer of E2F-driven gene expression and G1/S-transition in cancer cells and tumors. In consequence, IGF2BP1 promotes tumor cell proliferation *in vitro* and tumor growth *in vivo*. The concisely observed, proliferation-stimulating role of IGF2BP1 was largely attributed to the m^6^A-dependent stabilization of the MYC mRNA (9,10,12). However, IGF2BP1 and MYC mRNA expression appear barely correlated in most cancers and IGF2BP1 ablation results in conserved and exclusive impairment of G1/S transition. In contrast, disturbed cell cycle progression upon MYC depletion is variable, primarily leading to the enrichment of cancer cells in the S or G2/M phases (37). This clearly indicates that the specific role of IGF2BP1 in promoting G1/S-transition involves additional effector pathways controlling this checkpoint. We reveal that IGF2BP1 is a conserved regulator of E2F-driven gene expression, promoting the expression of E2F transcription factors and other positive regulators of G1/S transition like CDK2/4/6 as well as CCNE1 in an RNA-binding dependent manner. The only prior reported RBPs controlling E2F expression are PUM1 and 2, which promote miRNA-dependent repression of E2F3 mRNA translation (17,20). In contrast, IGF2BP1 impairs downregulation of the E2F1 mRNA by miRNAs. This unravels the first, conserved post-transcriptional enhancer of E2F-dependent cell cycle progression. The IGF2BP1-directed inhibition of target mRNA downregulation by miRNAs relies on their recruitment to miRNA/RISC-devoid IGF2BP1-mRNPs (14). The expression of positive regulators of G1/S transition, including E2Fs, is substantially impaired by abundant miRNAs emphasizing the potency of post-transcriptional control of cell cycle progression in cancer and stem cells (17,19,38). In addition to E2F1, IGF2BP1 directly stabilizes E2F-driven transcripts encoding cell cycle regulators like Ki-67. This identifies IGF2BP1 as a conserved post-transcriptional super-enhancer of E2F-transcritpion, promoting E2F activity over the cell type and mitogen-dependent restriction point of G1/S transition (17). Thus, our studies also provide an explanation for the conserved role of IGF2BPs in promoting the self-renewal of stem-like cells (2,39). E2F-driven transcription serves multiple roles in stem cells, including potentially cell cycle-independent regulation (18). Intriguingly, however, the ‘cell cycle length hypothesis’ implies that expanded time spent in the G1 phase increases the probability of guidance cues to induce differentiation of progenitor cells (40). Consistently, the overexpression of CDK4/cyclinD1 shortens G1 and promotes both, the generation and expansion of neural stem cells (41). The opposite, a substantial prolongation of G1, is observed when ablating IGF2BP1 expression. This suggests that IGF2BP1 favors an undifferentiated, stem-like cell phenotype with high self-renewal potential by shortening G1 due to enhanced G1/S transition. This is concise with IGF2BP1’s broad expression in cancer cell lines and substantial upregulation in progressed cancers. In these, the protein conveys elevated proliferation, self-renewal potential and anchorage-independent growth along with enforced expression of stem cell factors like LIN28B (8,14,42). A key observation of our study is that IGF2BP1/E2F-controlled cell cycle progression is apparently m^6^A-dependent. This emphasizes and substantially expands the recently reported m^6^A-reader role of IGF2BP1 (12,16). Far more important, however, these findings provide a mechanistic rational explaining the conserved growth-promoting role of METTL3/14 in cancer models, that remained controversial (43). Our study strongly suggests that METTL3/14-directed m^6^A-modification is a conserved mechanism promoting elevated cell cycle progression in IGF2BP1-expressing cancers. The enforcement of tumor cell proliferation is further amplified by the IGF2BP1-dependent stabilization of some E2F-driven mRNAs, encoding regulators of proliferation like Ki-67. This post-transcriptional ‘super’-enhancer function of IGF2BP1 is impaired by the small molecule BTYNB. The reported inhibition of IGF2BP1-RNA association and tumor cell proliferation by BTYNB impairing suggested that IGF2BP1-driven tumorigenesis is druggable in principal (21). Notably, however, MYC expression remained largely unchanged at BTYNB concentrations sufficient to substantially impair E2F expression and cell vitality. Moreover, putative off-target effects of BTYNB remain unknown so far. Nonetheless, we present evidence that BTYNB impairs IGF2BP1-dependent stabilization of mRNAs encoding factors, which promote cell cycle and cancer progression. The severe inhibition of tumor growth and peritoneal spread by BTYNB demonstrated in experimental tumor models provides strong, pre-clinical evidence that the therapeutic inhibition of IGF2BP1 is feasible. The conserved roles of IGF2BP1 and inhibitory potency of BTYNB revealed here suggest a broad therapeutic target potential of IGF2BP1 in the treatment of solid cancers. Furthermore, BTYNB acts in an additive manner with Palbociclib, a cell cycle inhibitor targeting key E2F-activaiting kinases, mainly CDK4 and CDK6 (36). Thus, our studies provide pre-clinical evidence that combined treatment with BTYNB, impairing IGF2BP1-RNA association, is beneficial for cell cycle inhibition, e.g. by Palbociclib, in cancer treatment.

## DATA AVAILABILITY

RNA-sequencing data are deposited on GEO: GSE146807

Flow cytometry data are deposited on FLOW Repository: FR-FCM-Z2DE, FR-FCM-Z2DG, FR-FCM-Z2DF, FR-FCM-Z2ET, FR-FCM-Z2DW

## Supplementary Material

gkaa653_Supplemental_Files
