# Supplementary material for: The oncofetal RNA-binding protein IGF2BP1 is a druggable, post-transcriptional super-enhancer of E2F-driven gene expression in cancer
Source: Nucleic Acids Res. 2020 Aug 6;48(15):8576–90. doi: 10.1093/nar/gkaa653 (PMC7470957; doi:10.1093/nar/gkaa653)

SUPPLEMENTARY FIGURE LEGENDS

Supplementary Figure S1 - IGF2BP1 is a conserved oncoRBP in solid human cancer. (**A)** Heatmap of IGF2BP1 Hazard ratios (HR) with indicated p-values. Overall survival (median cutoff) was analyzed for indicated TCGA tumor cohorts. (**B)** Heatmap showing the $\log_2$ FC of mRBP-encoding mRNAs (n=660, left heatmap) in indicated TCGA tumor cohorts to corresponding GTEx normal tissue samples based on RNA sequencing. mRBPs were ranked according to their average FC. The $\log_2$ FC of the 10 most upregulated mRBPs is depicted by a heatmap enlargement (right panel). **(C, D)** Scatter plots of IGF2BP1 and MYC expression across all TCGA tumor cohorts (n =33, C) and five indicated TCGA cancer cohorts (D).

Supplementary Figure S2 - IGF2BP1 expression shows conserved association with enhanced proliferation in solid cancer. **(A)** Violin plots of correlation coefficients (R) determined for protein-coding genes and IGF2BP1 expression in the indicated TCGA tumor cohorts. **(B, C)** Gene set enrichment analysis (GSEA) of IGF2BP1-correlated gene expression in the five indicated TCGA tumor cohorts. GSEA was performed on the ranked correlation coefficients determined in **A**. GSEA plots for indicated cancer cohorts are shown for the cancer Hallmark pathway “E2F targets” (**B**) and KEGG pathway “Cell Cycle” (**C**). NES, normalized enrichment score.

Supplementary Figure S3 - IGF2BP1 is a conserved regulator of tumor cell proliferation and clonogenic growth. **(A-C)** PANC-1 cells were transfected with control (siCtrl, grey) or IGF2BP1-directed siRNA pools (siIGF2BP1, blue). Cells were counted at indicated time points post-transfection by flow cytometry (**A**) and the doubling time was calculated (**B**) in three independent experiments. The percentage of Propidium iodide (PI) positive and negative PANC-1 cells (**C**) was determined by flow cytometry upon transfection of indicated siRNAs. **(D, E)** Quantification of colony formation and clonogenic growth of PANC-1 cells transfected with indicated siRNAs. Representative images of colony formation in soft agar (**D**, left panel) and clonogenicity studies (**E**, left panel) are shown. Statistical significance was determined by Mann-Whitney-Test: *, $p<0.05$; **, $p< 0.01$; ***, $p< 0.001$.

Supplementary Figure S4 - IGF2BP1 is a conserved enhancer of G1/S transition in cancer-derived cells. **(A)** Representative cell cycle phase distribution upon transfection with control (siCtrl) or IGF2BP1-directed siRNAs in indicated cancer cells, as determined by PI-labeling and flow cytometry. **(B, C)** ES-2 cells were stably transduced with the FUCCI system (Sartorius) and transfected with control (siCtrl) or IGF2BP1-directed siRNAs. Cells were synchronized in G2 phase (green fluorescence) 20h post-transfection by cell sorting (FACS) and monitored for indicated time in the IncuCyte Live Cell Imaging

system. Cell segmentation and classification was performed using the Cell-by-Cell module and representative images overlayed with segmentation masks are shown (**B**). Difference between IGF2BP1 depleted and control cells is shown for the respective cell cycle phases over time for n > 4000 objects (**C**). Error bars indicate standard error of three independent experiments. Red, G1 phase; Green, G2 phase; Yellow, S phase. **(D)** Spheroid growth of parental (Ctrl) and IGF2BP1-deleted (KO) A549 cells. Representative A549 spheroids 5 days post-seeding are indicated in the left panel. The viability of spheroids was determined by Cell-titer GLO (right panel). **(E)** Spheroid growth analysis of IGF2BP1-KO A549 cells expressing GFP, GFP-IGF2BP1 (WT) or an RNA-binding deficient GFP-IGF2BP1 (MUT). Spheroid viability was determined using Cell Titer GLO and normalized to median cell vitality observed in GFP-expressing controls. Error bars indicate standard deviation of at least three independent analyses with at least four analyzed spheroids. Statistical significance was determined by Mann-Whitney-Test.

Supplementary Figure S5 - IGF2BP1 is a conserved regulator of E2F-driven gene expression in cancer-derived cells. **(A)** Gene set enrichment analysis (GSEA) of protein-coding gene fold changes upon IGF2BP1 depletion in five indicated cancer cell lines the median fold change determined in the respective analyses as shown in Figure 3. GSEA plots for the Hallmark pathway “E2F Targets” (top panel) and the KEGG pathway “Cell Cycle” (bottom panel) are shown. NES, normalized enrichment score. **(B)** IGF2BP1 CLIP profile of the indicated mRNAs. The 3’UTR is highlighted by dashed lines. **(C-E)** Representative Western blot analyses of E2F2 and E2F3 protein expression upon IGF2BP1-depletion in PANC-1 cells (C), of E2F1 protein upon IGF2BP1 deletion in A549 (D) cells by CRISPR/Cas9 and of E2F1 protein in IGF2BP1-KO A549 cells expressing GFP, GFP-IGF2BP1 (WT) or an RNA-binding deficient GFP-IGF2BP1 (MUT) (E). Vinculin (VCL) served as loading and normalization control. Average fold changes and standard deviation of E2F1/2/3 protein levels, determined in three independent analyses are indicated in bottom panels. **(E)** Scatter Plots of IGF2BP1, METTL3, METTL14 (x-axis) and E2F1, E2F2, E2F3 (y-axis and in plot) expression across all TCGA tumor cohorts (33 cohorts, 9282 patients) determined by GEPIA2. Pearson correlation coefficient and p-value are indicated.

Supplementary Figure S6 – IGF2BP1 controls E2F1 mRNA abundance in a miRNA- and m^6A-dependent manner. **(A)** RT-qPCR analysis of indicated mRNA levels in total RNA (blue) or nascent RNA (grey) fractions upon IGF2BP1-depletion normalized to control-transfected PANC-1 cells. RPLP0 served as normalization control. Nascent RNAs were purified using the Click-iT Nascent RNA Capture Kit (Thermo Fisher) upon labeling newly synthesized transcripts with 5-ethynyl uridine for 4h (72h post-transfection of siRNAs). **(B)** Abundance of miRNAs targeting the E2F1 3’UTR. MiRNA expression, determined by

small RNA sequencing in five cancer cell lines, is indicated as median cpm along with the number of 12 investigated databases predicting miRNA binding sites (MBS), as analyzed by miRWalk2.0. **(C)** Luciferase reporter analysis demonstrating activity of indicated reporters in control- (siCtrl, red) or IGF2BP1-depleted (siIGF2BP1, blue) A549 cells. Reporter activities, normalized to a control reporter without miRNA targeting site (Empty), were determined in three independent experiments with two technical replicates each. **(D)** Immunoprecipitation of AGO2 in parental (Ctrl) or IGF2BP1-knockout A549 cells was confirmed by Western blotting (left panel). VCL served as negative control. **(E, F)** Representative Western blot analyses of E2F2 and E2F3 proteins upon METTL3 and METTL14-depletion in PANC-1 cells (E) and of E2F1 protein upon METTL3 deletion (F) in A549 by CRISPR/Cas9. Vinculin (VCL) served as loading and normalization control. Average fold changes and standard deviation of E2F1/2/3 protein levels, determined in three independent analyses are indicated in bottom panels. **(G)** Scatter Plots of IGF2BP1, METTL3, METTL14 (x-axis) and E2F1, E2F2, E2F3 (y-axis and in plot) expression across all TCGA tumor cohorts (33 cohorts, 9282 patients) determined by GEPIA2. Pearson correlation coefficient and p-value are indicated.

Supplementary Figure S7 - IGF2BP1 is a post-transcriptional super-enhancer of E2F-driven gene expression in cancer. **(A)** IGF2BP1 CLIP profile in the 3'UTR of the indicated E2F-driven mRNAs. The length of the 3'UTRs is shown. **(B, C)** PANC-1 cells were transfected with control (siC, black) or E2F1/2/3-directed siRNA pools (siE2F1+2+3, blue). The viability of 2D-cultured PANC-1 cells (**B**) and PANC-1 spheroids (**C**, right panel) was determined by Cell-titer GLO 6 days post-transfection. Representative PANC-1 spheroids are shown in the left panel (**c**). Statistical significance was determined by Mann-Whitney-Test: **, $p < 0.01$; ***, $p < 0.001$. **(D)** Scatter Plots of IGF2BP1 (x-axis) and indicated mRNAs (y-axis and in plot) expression across all TCGA tumor cohorts (33 cohorts, 9282 patients, top panel) and the LIHC, LUAD, OV, SKCM and PAAD cohorts (bottom panel) determined by GEPIA2. The correlation of IGF2BP1 and the 31 gene signature shown in Figure 6C is depicted in the right scatter plot. Correlation coefficients (R) and p-values are indicated in plots.

Supplementary Figure S8 - BTYNB is a potent inhibitor of IGF2BP1-driven tumor cell proliferation and tumor growth. **(A)** Indicated cancer cells were treated with DMSO (grey) or 5 µM BTYNB (blue) for 48 h. Representative images of PANC-1 cells are indicated in the left panel. Cell viability was determined by Cell-titer GLO (right panel). **(B)** Immunoprecipitation of IGF2BP1 in A549 cells treated with DMSO, 5 µM BTYNB or 5µM Palbociclib for 24h was confirmed by Western blotting. VCL served as negative control. **(C, D)** Co-purification of indicated mRNAs with IGF2BP1 in DMSO-, Palbociclib-treated (5µM for 24h, C) or BTYNB-treated (5µM for 24h, D) A549 cells was analyzed by RIP using anti-IGF2BP1 antibodies and RT-qPCR analysis. The enrichment of mRNAs in BTYNB-treated over DMSO-treated cells

is shown. HIST1 mRNA served as normalization control. **(E)** RT-q-PCR analysis of mRNA levels upon BTYNB treatment in A549 cells normalized to DMSO-treated cells as in Figure 7A-D. RPLP0 served as internal normalization control. **(F, G)** iRFP-labeled ES-2 cells were treated with DMSO or 5 µM BTYNB for 24h and upon s.c. (F) or i.p. (G) injection into nude mice (5 mice per condition). Representative images indicating iRFP-labeled tumors at indicated times are shown in the left panels. Quantifications of tumor volumes (F) or fluorescence intensity (FI, G) are shown in the right panels. (**G**, bottom panel) Arrows indicate i.p. tumors 14 days post-injection in an enlargement of the region indicated by a dashed box in the middle panel. Statistical significance was determined by Mann-Whitney-Test. Exact p-values are indicated.

Supplementary Figure S1

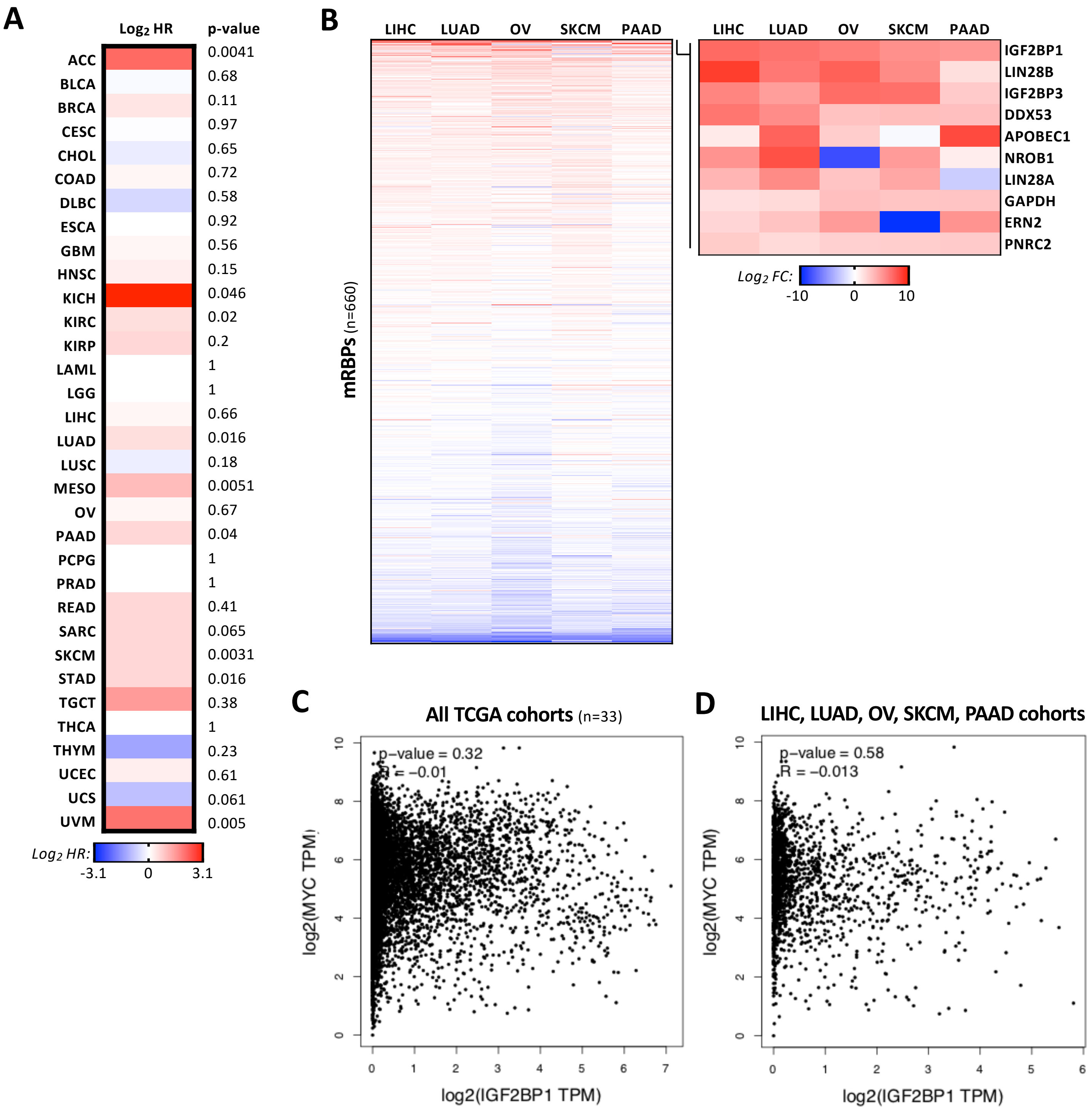

Supplementary Figure S2

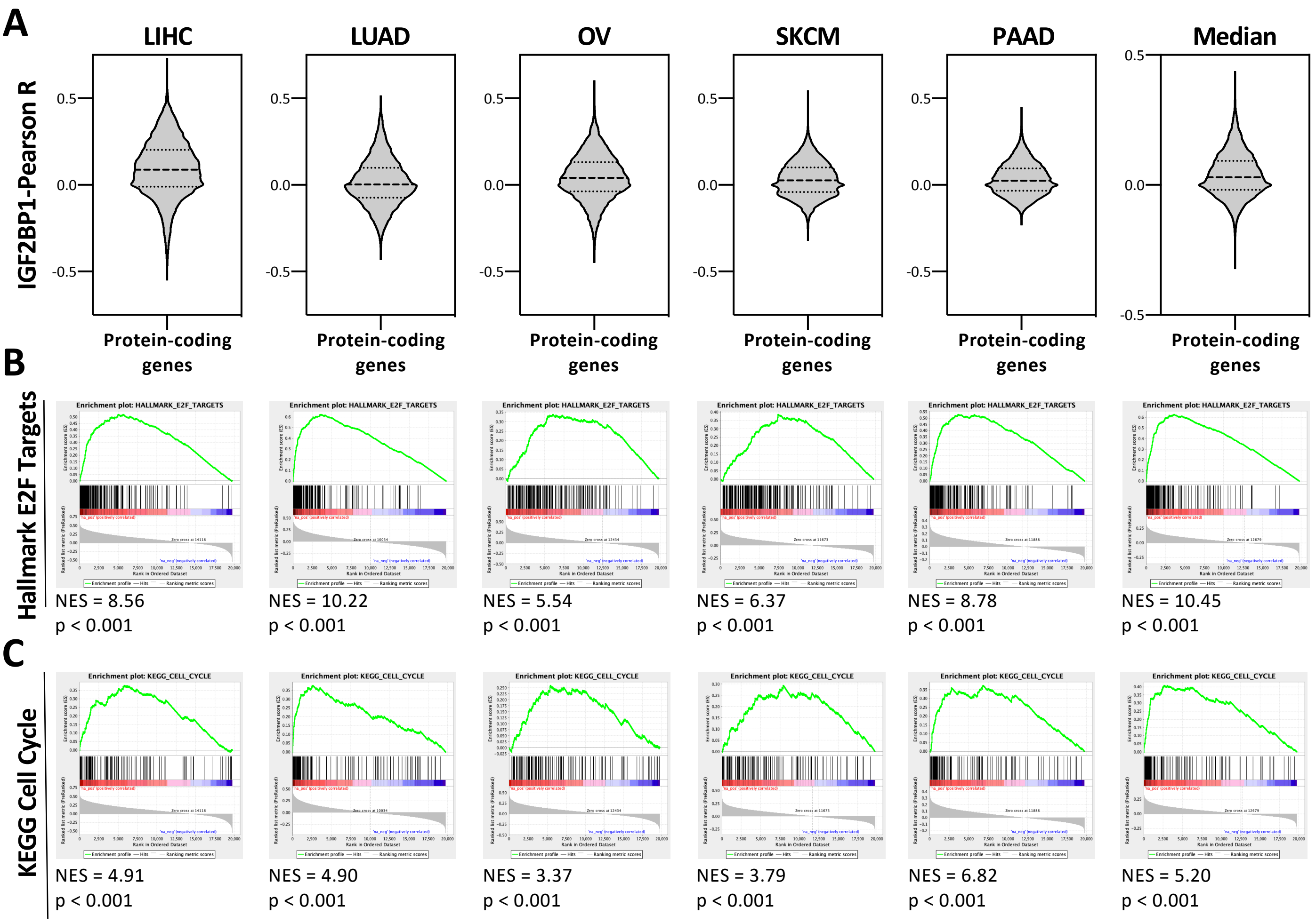

Supplementary Figure S3

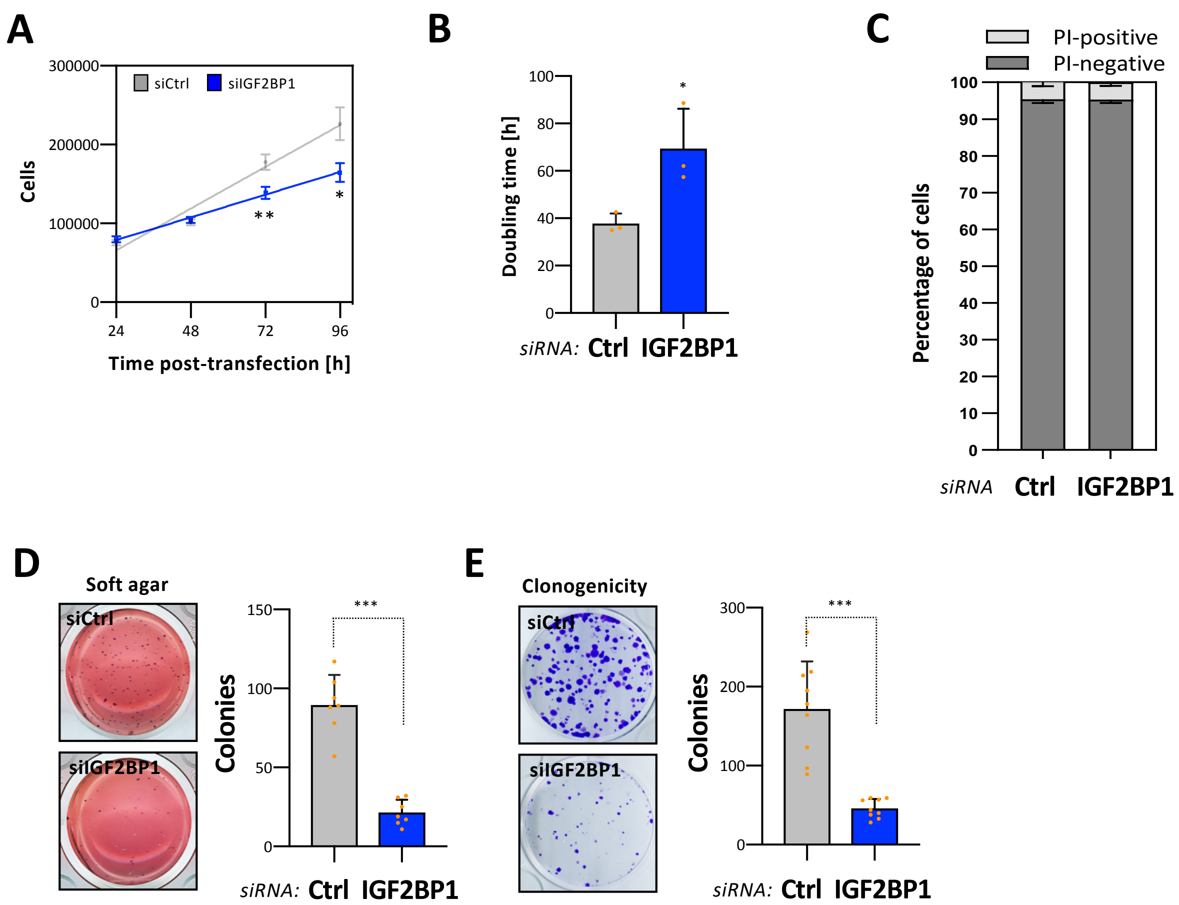

Supplementary Figure S4

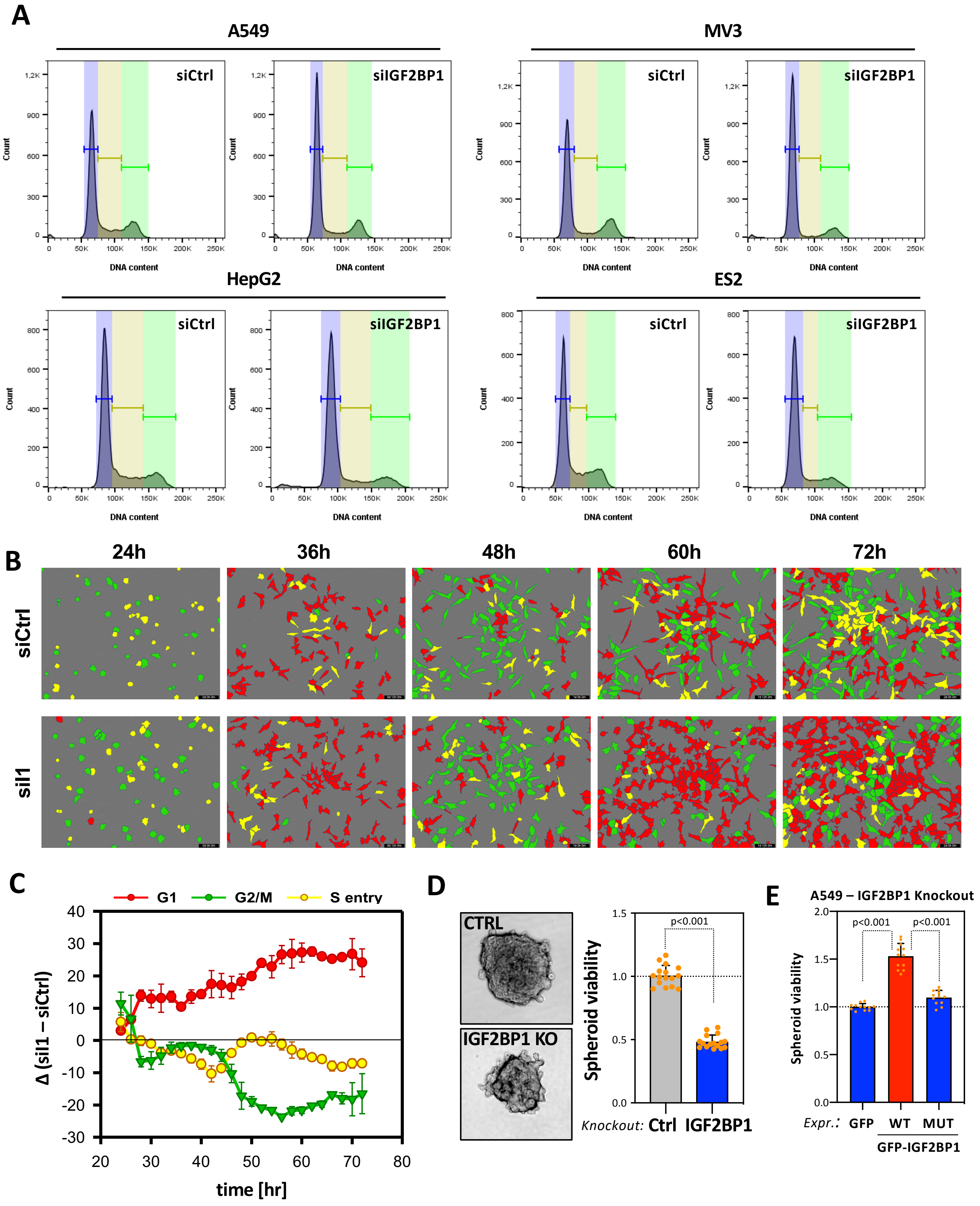

Supplementary Figure S5

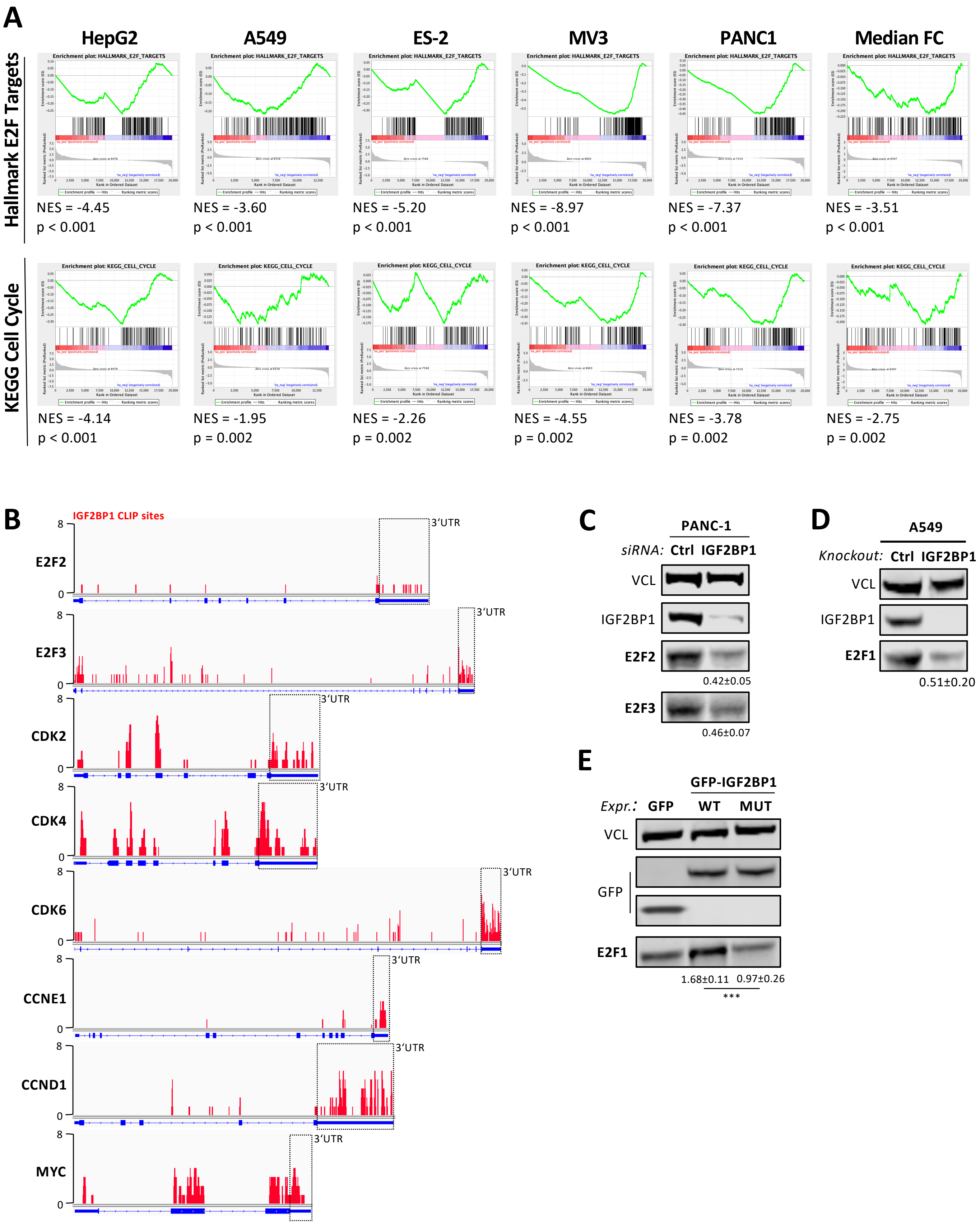

Supplementary Figure S6

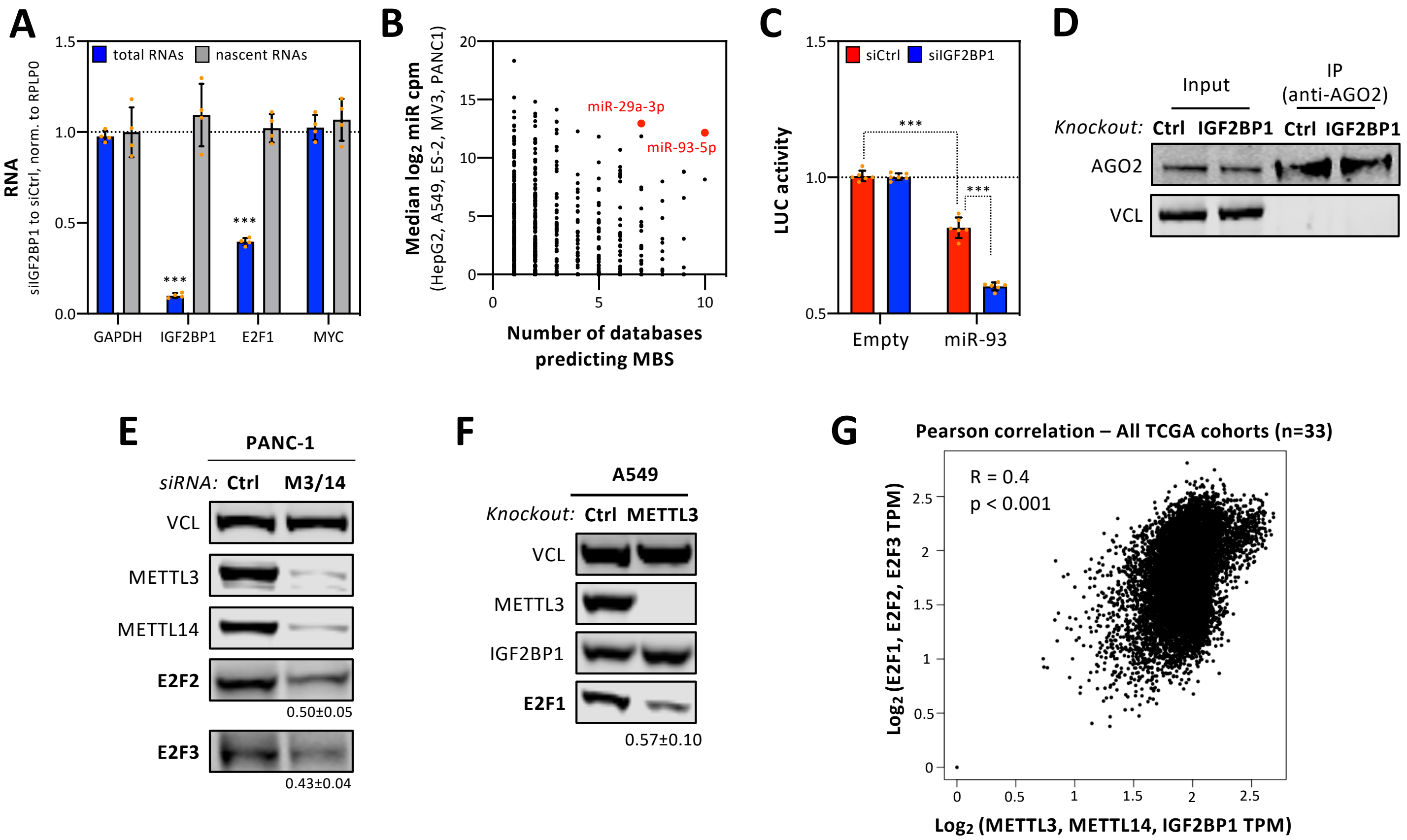

Supplementary Figure S7

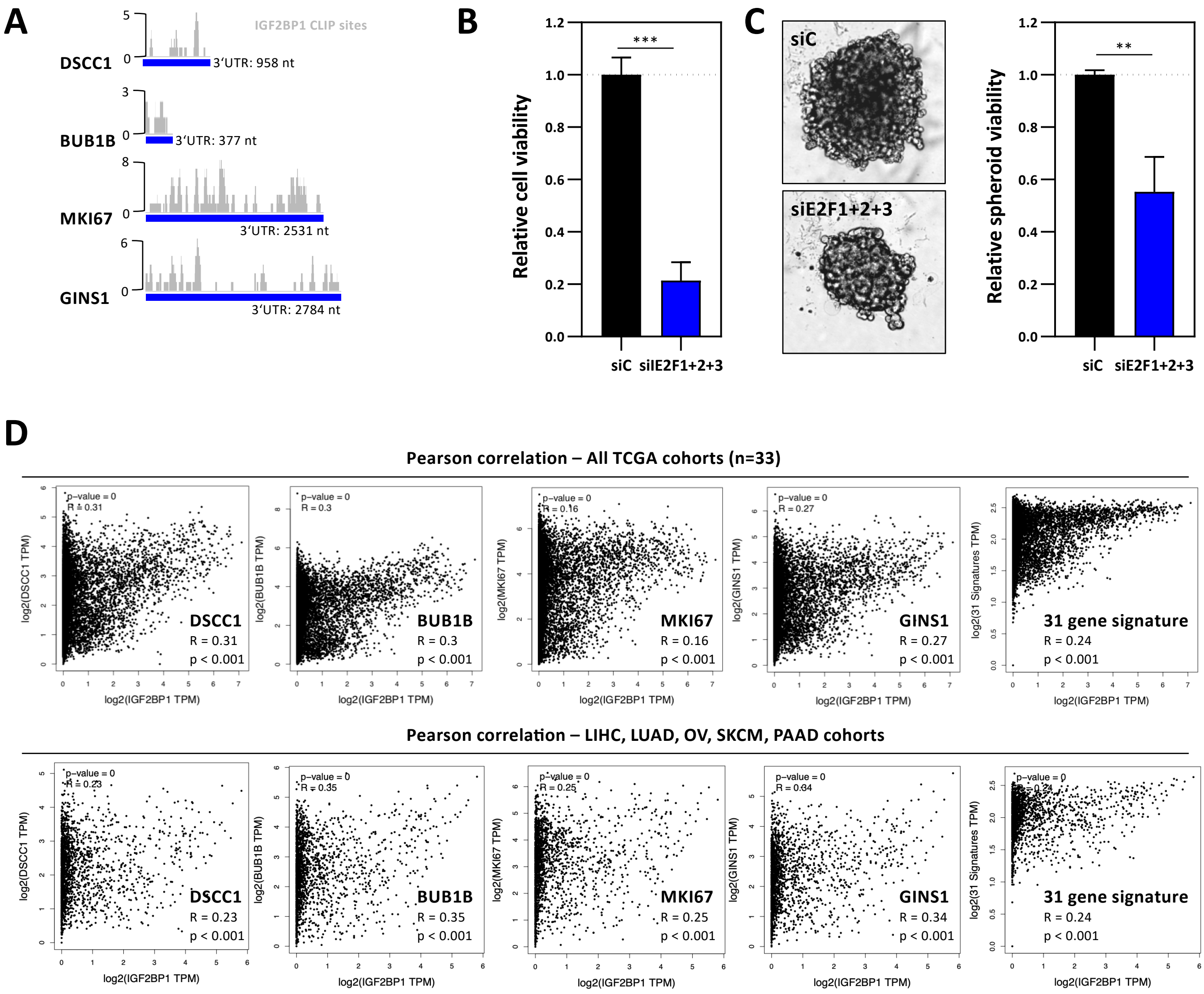

Supplementary Figure S8

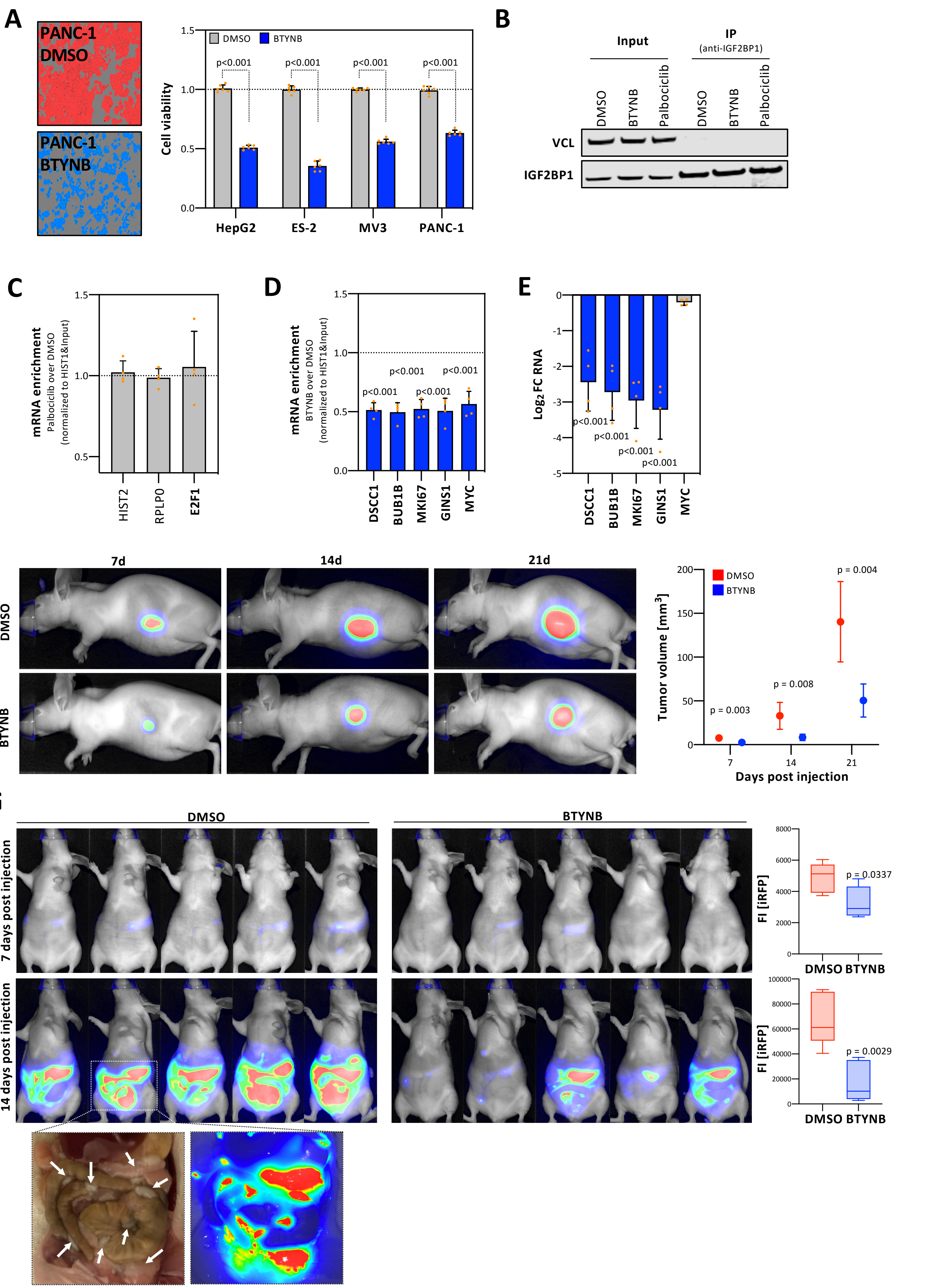

Supplement: gkaa653_Supplemental_Files [file gkaa653_supplemental_files.zip › NAR_Supplementary Figures Legends_rev1.pdf]
